# GUTK induces apoptosis in reactivating quiescent prostate cancer cells *via* Aurora A-mediated stabilization of SOD2

**DOI:** 10.1016/j.isci.2026.115739

**Published:** 2026-04-15

**Authors:** Yalin Wang, Yang Li, Xue Jiang, Xiaoqiong Chen, Mengfan Liu, Hangui Ren, Yulong Zhang, Rongchen Dai, Zhichao Xi, Hongxi Xu

**Affiliations:** 1 School of Pharmacy, Shanghai University of Traditional Chinese Medicine, Shanghai 201203, China; 2 Engineering Research Center of Shanghai Colleges for TCM New Drug Discovery, Shanghai 201203, China; 3 Yueyang Hospital of Integrated Traditional Chinese and Western Medicine, Shanghai University of Traditional Chinese Medicine, Shanghai 200437, China

**Keywords:** pharmacology, biochemistry, cancer

## Abstract

Quiescent prostate cancer (PCa) cells that survive therapy can later reactivate and drive tumor recurrence and metastasis. Here, we identify a strategy to eliminate these cells during their vulnerable reactivation phase. We show that guttiferone K (GUTK), a bioactive compound isolated from *Garcinia yunnanensis* Hu, selectively eradicates reactivating quiescent PCa cells by inducing mitochondrial apoptosis through caspase activation and loss of mitochondrial membrane potential (ΔΨm). Mechanistically, GUTK suppresses Aurora A recovery and stabilizes SOD2 protein, thereby promoting mitochondrial dysfunction and apoptosis. SOD2 enhances, whereas Aurora A overexpression attenuates, GUTK-induced cell death. In orthotopic and xenograft prostate tumor models, GUTK combined with docetaxel significantly inhibits tumor growth and suppresses post-chemotherapy recurrence without evident toxicity. These findings identify GUTK as a potential therapeutic agent targeting reactivating quiescent PCa cells and highlight the Aurora A-SOD2 axis as a promising pathway for preventing PCa recurrence.

## Highlights

• GUTK selectively eliminates reactivating quiescent PCa cells
• GUTK induces mitochondrial apoptosis via caspase activation and ΔΨm loss
• GUTK suppresses Aurora A and stabilizes SOD2 to promote apoptosis
• Aurora A-SOD2 axis is a potential target to prevent recurrence from quiescent cells

## Graphical abstract

**Figure:**
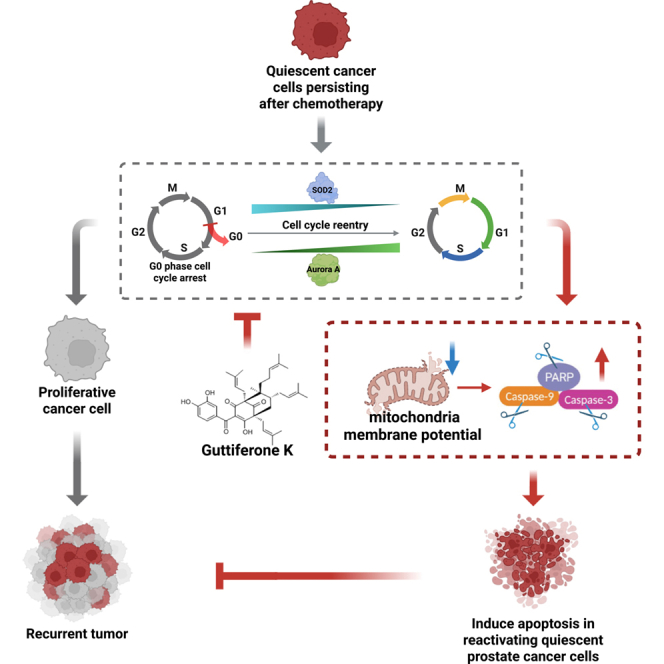

## Introduction

Prostate cancer (PCa) remains a leading cause of cancer-related mortality worldwide.^1^ Although effective treatments such as androgen deprivation therapy (ADT), radiation therapy, and radical prostatectomy can control the primary disease, mortality rates remain high due to therapeutic resistance and metastatic recurrence.^2^ The presence of quiescent PCa cells is considered a critical barrier to cure, a dormant subpopulation that persists in a reversible G0 state, exhibiting characteristics such as enhanced drug resistance, stem cell-like properties, and abnormal metabolism.^3^ While cytotoxic agents such as docetaxel effectively kill proliferating cancer cells, they fail to eradicate quiescent populations.^4^ Consequently, these residual cells can re-enter the cell cycle and drive tumor relapse. Notably, there are currently no effective drugs available that specifically target quiescent cancer cells to prevent cancer recurrence.^5^ Therefore, identifying compounds that selectively eliminate quiescent PCa cells during reactivation represents an urgent therapeutic imperative.^6^

Mitochondrial integrity and function govern cell fate decisions during reactivation, and preserving mitochondrial balance is crucial for the survival of quiescent cancer cells.^7^^,^^8^ The mitochondrial matrix enzyme superoxide dismutase 2 (SOD2) has been identified as aberrantly overexpressed in quiescent PCa cells, suggesting a significant role in tumor dormancy.^9^ SOD2 primarily maintains redox balance by converting superoxide anion radicals to H_2_O_2_ and O_2_.^10^^,^^11^ Although SOD2 is conventionally characterized as a protective antioxidant, emerging evidence indicates that it plays a context-dependent role in quiescent cells. For example, elevated SOD2 facilitates quiescence entry in fibroblasts *via* metabolic reprogramming.^12^ Paradoxically, our previous research demonstrated that SOD2 overexpression induced apoptosis in awakening quiescent PCa cells, while its knockdown accelerated cell cycle re-entry, indicating a detrimental role of SOD2 during quiescent cancer cell reactivation.^9^ Therefore, targeting this unique, context-specific pro-death function of SOD2 in awakening quiescent cancer cells represents a promising strategy for developing novel therapeutics to prevent PCa relapse.

Aurora kinase A (encoded by AURKA) orchestrates mitotic spindle assembly and activates regulators such as Polo-like kinase 1 and Cyclin B-cyclin-dependent kinase 1, while suppressing tumor suppressors such as p53.^13^^,^^14^^,^^15^ It is often overexpressed in cancers, driving genomic instability and chemoresistance.^16^^,^^17^^,^^18^ Intriguingly, bioinformatic and functional studies identify SOD2 as a potential Aurora A ubiquitination substrate. Aurora A binds SOD2 and promotes its K48-linked degradation, whereas Aurora A inhibition elevates SOD2, leading to mitochondrial dysfunction.^19^ However, whether these pathways are involved in quiescent PCa cell survival is unknown.

Natural products offer promising scaffolds for eliminating therapy-resistant quiescent cancer cells.^20^ Increasing research is underway on drugs targeting quiescent cancer cells, encompassing both clinically available agents and those under preclinical investigation.^21^ Current paradigms for targeting quiescent cancer cells are largely divided into three categories: enforcing dormancy, direct killing, or reactivation to sensitize cells to conventional therapies. Current direct elimination strategies face a significant hurdle: the need to identify unique and consistently targetable vulnerabilities within the dormant state itself, which are often elusive. Here, we describe a novel strategy that overcomes this challenge. We show that the natural compound guttiferone K (GUTK) does not merely directly kill quiescent cancer cells nor simply reactivate them; Instead, it exploits the reactivation process to induce apoptosis. This mechanism initiates reactivation and simultaneously triggers cell death during this transition. This represents a significant conceptual advance and provides a potent strategy to eliminate residual cancer cells and prevent relapse.

In this study, we revealed that GUTK induces mitochondrial dysfunction and caspase-dependent apoptosis in quiescent PCa cells during reactivation. Mechanistically, GUTK upregulates SOD2 protein, and SOD2 knockdown weakens GUTK-induced apoptosis and mitochondrial membrane potential (ΔΨm) loss. SOD2 overexpression exacerbates cytotoxicity and directly induces cell death in quiescent PCa cells during reactivation. Aurora A overexpression downregulates SOD2 protein levels and reverses GUTK-induced apoptosis, suggesting that Aurora A functions as an upstream regulator of SOD2. Moreover, GUTK combined with docetaxel enhanced the reduction of orthotopic prostate tumors *in vivo* without causing obvious toxicity. Our findings suggest that GUTK is a promising candidate for triggering mitochondrial apoptosis in awakening quiescent PCa cells and highlight the Aurora A-SOD2 pathway as a potential therapeutic target for eliminating quiescent cancer cells during reactivation.

## Results

### GUTK induces mitochondrial dysfunction and caspase-dependent apoptosis to eliminate quiescent PCa cells during reactivation

**Figure 1:**
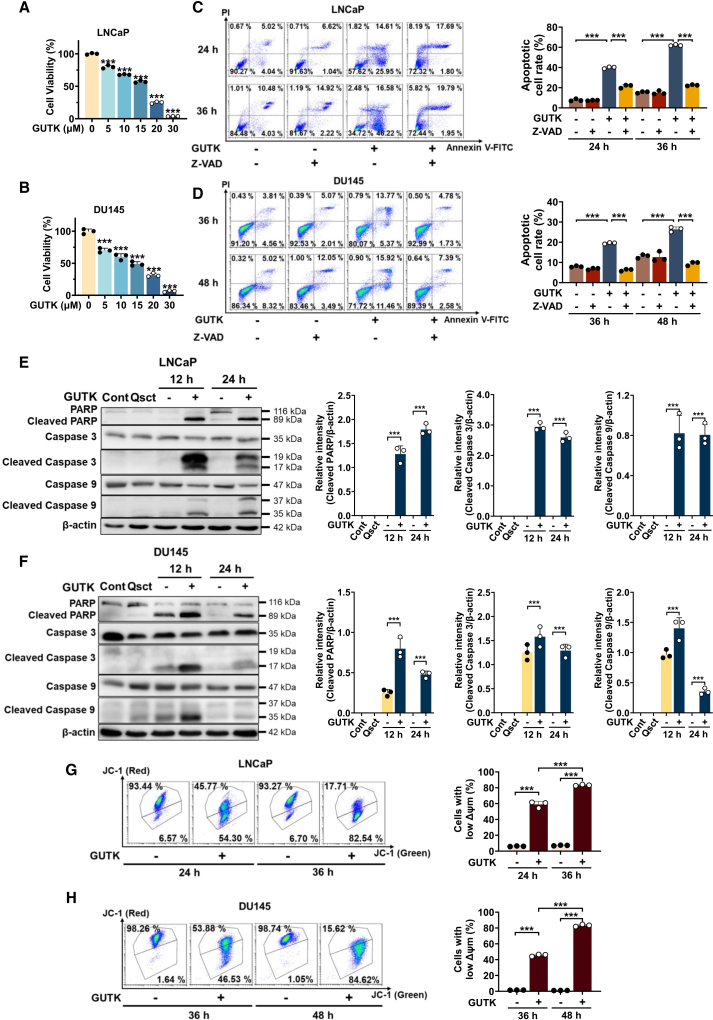
GUTK induces apoptosis and ΔΨm loss in quiescent PCa cells during cell cycle re-entry (A and B) Cell viability assessed by CCK-8 assay in quiescent LNCaP (A) and DU145 (B) cells treated with the indicated concentrations of GUTK for 48 h during reactivation. (C and D) Apoptosis analysis by Annexin V-FITC/PI staining and flow cytometry in quiescent LNCaP (C) and DU145 (D) cells treated with 20 μM GUTK alone or co-treated with 50 μM Z-VAD during cell cycle re-entry. Quantification of apoptotic cells is shown in the right panel. (E and F) Immunoblot analysis of apoptosis-related proteins (PARP, caspase 3, caspase 9, and their cleaved forms) in quiescent LNCaP (E) and DU145 (F) cells treated with 20 μM GUTK for 12 h or 24 h during cell cycle re-entry. β-actin served as the loading control. Band intensity was quantified using ImageJ software (right panel). (G and H) ΔΨm loss assessed by JC-1 staining and flow cytometry in quiescent LNCaP (G) and DU145 (H) cells treated with 20 μM GUTK at the indicated times during cell cycle re-entry. Quantitative data show the percentages of cells with low ΔΨm at specified times (right panel). Cont: proliferating control cells; Qsct: quiescent cells. Data are presented as mean ± SD from three independent experiments. ∗∗∗*p* < 0.001 versus the indicated group.

To determine whether GUTK eradicates awakening quiescent PCa cells, we first assessed cell viability 48 h after treatment with GUTK (0–30 μM) during reactivation. A CCK-8 assay revealed a concentration-dependent decline in viability in both LNCaP (Figure 1A) and DU145 (Figure 1B) cells. Notably, doses above 20 μM reduced viability by more than 60%, indicating that GUTK induces cell death in awakening quiescent PCa cells.

To elucidate the mode of cell death, we performed Annexin V/PI double staining followed by flow cytometry. In LNCaP cells treated with 20 μM GUTK, apoptosis rates reached 40% at 24 h and 62% at 36 h post-reactivation (Figure 1C). In DU145 cells, apoptotic cells accounted for 19% at 36 h and 27% at 48 h post-reactivation (Figure 1D). Importantly, co-treatment with the pan-caspase inhibitor Z-VAD reduced GUTK-induced apoptosis by more than 50% (Figures 1C and 1D). Consistent with these findings, immunoblot analysis revealed significant increases in cleaved PARP, cleaved caspase 3 and cleaved caspase 9 in GUTK-treated cells during reactivation (Figures 1E and 1F), indicating that caspase-dependent apoptosis is a primary mechanism of GUTK-triggered cell death.

Furthermore, GUTK significantly decreased ΔΨm in both awakening quiescent LNCaP (Figure 1G) and DU145 (Figure 1H) cells. Specifically, treatment with 20 μM GUTK for 24 h and 36 h post-reactivation increased the fraction of LNCaP and DU145 cells with low ΔΨm from ∼5% to 50%. This effect intensified at 36 h post-reactivation, with over 80% of cells exhibiting low ΔΨm. Together, these data demonstrate that GUTK disrupts mitochondrial function and induces caspase-dependent apoptosis, thereby blocking cell cycle re-entry in quiescent PCa cells.

### GUTK induces apoptosis largely independent of androgen receptor signaling during PCa cell reactivation

As LNCaP cells are an androgen-sensitive human PCa cell line, we investigated whether androgen receptor (AR) signaling plays a critical role in their re-proliferation from quiescence. Reactivating quiescent LNCaP cells exhibited a dose-dependent reduction in viability upon treatment with AR inhibitors enzalutamide (ENZ) and apalutamide (APL) (Figure S1A), with no significant difference in sensitivity compared with proliferative LNCaP cells (Figure S1B). These results indicate that the survival of LNCaP cells remains dependent on AR signaling even during reactivation from quiescence.

Consistent with this observation, AR expression decreased during quiescence and was restored upon reactivation, whereas treatment with the potent synthetic AR agonist metribolone (R1881) further enhanced AR expression throughout this process (Figure S1C). Although R1881 alone promoted cell viability during reactivation, GUTK robustly induced cell death even in the presence of R1881 (Figure S1E), without altering AR protein expression (Figure S1D). Moreover, GUTK induced apoptosis in both reactivating quiescent LNCaP (AR-positive) cells and DU145 (AR-negative) cells. Collectively, these findings indicate that GUTK induces apoptosis during PCa cell reactivation through a mechanism that is largely independent of AR signaling, although the precise downstream pathways mediating this effect require further investigation.

### GUTK induces apoptosis *via* sustaining SOD2 protein levels during the reactivation of PCa cells

**Figure 2:**
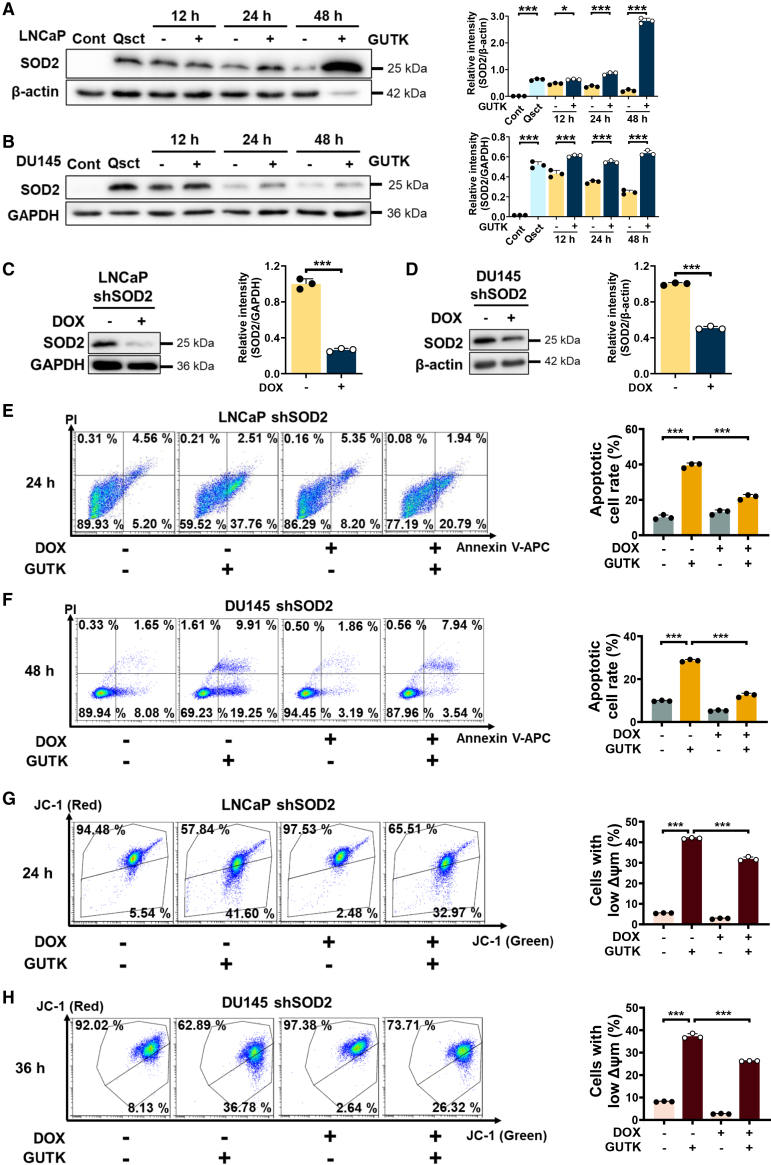
SOD2 knockdown rescues GUTK-induced apoptosis and ΔΨm loss in reactivating quiescent PCa cells (A and B) Immunoblot analysis of SOD2 protein levels in quiescent LNCaP (A) and DU145 (B) cells treated with 20 μM GUTK for 12–48 h during cell cycle re-entry. β-actin and GAPDH served as loading controls. Quantification of relative protein levels is shown in the right panel. (C and D) Validation of SOD2 knockdown by immunoblot analysis in LNCaP (C) and DU145 (D) cells transfected with shSOD2. β-actin or GAPDH served as loading controls. Quantification of protein levels is shown in the right panel. (E and F) LNCaP shSOD2 and DU145 shSOD2 cells were treated with or without DOX to induce SOD2 knockdown. Apoptosis was assessed by Annexin V-APC/PI staining and flow cytometry in LNCaP shSOD2 (E) and DU145 shSOD2 (F) cells treated with DMSO or 20 μM GUTK for the indicated times during cell cycle re-entry. Quantification of apoptotic cell percentage is shown in the right panel. (G and H) ΔΨm assessment by JC-1 staining and flow cytometry in quiescent LNCaP shSOD2 (G) and DU145 shSOD2 (H) cells treated with either DMSO or 20 μM GUTK for the indicated times during cell cycle re-entry. Quantification of cells with low ΔΨm is shown in the right panel. Cont: proliferating control cells; Qsct: quiescent cells. ΔΨm: mitochondrial membrane potential. Data are presented as mean ± SD from three independent experiments. ∗*p* < 0.05 and ∗∗∗*p* < 0.001 versus indicated group.

Our previous findings demonstrated that SOD2 plays a pivotal role in regulating cell survival during the reactivation of quiescent PCa cells, and that its upregulation may serve as a promising therapeutic strategy to prevent recurrence driven by these cells.^9^ We therefore investigated whether GUTK-induced apoptosis is associated with SOD2 upregulation. Immunoblot analysis confirmed that SOD2 protein levels were significantly higher in quiescent PCa cells compared to their proliferating counterparts and progressively declined upon reactivation. Notably, treatment with 20 μM GUTK sustained elevated SOD2 expression throughout the reactivation process in both LNCaP (Figure 2A) and DU145 (Figure 2B) cells at 12 h, 24 h, and 48 h, indicating that SOD2 may play a key role in GUTK-induced apoptosis.

To further determine whether SOD2 upregulation is essential for GUTK-induced apoptosis, we established stable cell lines expressing DOX-inducible SOD2 shRNA. Efficient knockdown of SOD2 at the protein level was confirmed by immunoblot analysis (Figures 2C and 2D). SOD2 silencing significantly reduced GUTK-induced apoptosis by approximately 50% in reactivating quiescent LNCaP (Figure 2E) and DU145 cells (Figure 2F). Correspondingly, GUTK-induced loss of ΔΨm in awakening quiescent LNCaP (Figure 2G) and DU145 cells (Figure 2H) was also significantly rescued by SOD2 knockdown. These findings underscore SOD2 as a critical mediator of GUTK-induced apoptosis during quiescent PCa cell reactivation.

### SOD2 overexpression eliminates reactivating PCa cells and enhances GUTK-induced apoptosis

**Figure 3:**
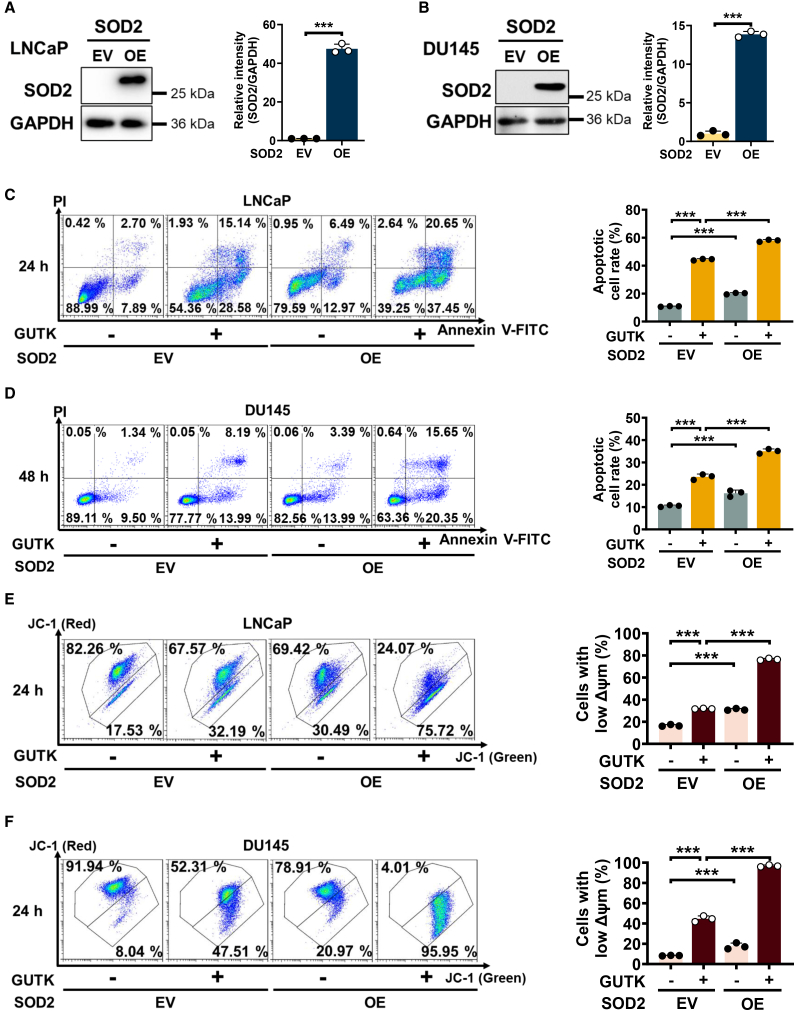
SOD2 overexpression exacerbates GUTK-induced apoptosis and ΔΨm loss in reactivating quiescent PCa cells (A and B) Immunoblot validation of SOD2 overexpression in LNCaP (A) and DU145 (B) cells stably transfected with empty vector (EV) or SOD2 expression construct (SOD2 OE). GAPDH served as the loading control. Quantification of relative protein levels is shown in the right panel. (C and D) Annexin V-FITC/PI staining and flow cytometry analysis of EV control or SOD2 OE LNCaP (C) and DU145 (D) cells treated with or without 20 μM GUTK for the indicated times during cell cycle re-entry. Quantification of apoptotic cell percentages is shown in the right panel. (E and F) ΔΨm analysis by JC-1 staining and flow cytometry in EV control or SOD2 OE LNCaP (E) and DU145 (F) cells treated with or without 20 μM GUTK for the indicated times during cell cycle re-entry. Quantification of cells with low ΔΨm is shown in the right panel. ΔΨm: mitochondrial membrane potential. Data are presented as mean ± SD from three independent experiments. ∗∗∗*p* < 0.001 versus indicated group.

To further elucidate the role of SOD2 in GUTK-induced apoptosis during quiescent PCa cell reactivation, LNCaP (Figure 3A) and DU145 (Figure 3B) cells were stably transfected with either full-length SOD2 cDNA (SOD2 overexpression, SOD OE) or an empty vector (EV) control. Consistent with our previous report,^9^ SOD2 overexpression induced substantial apoptosis (Figures 3C and 3D) and ΔΨm loss (Figures 3E and 3F) in reactivating quiescent PCa cells compared to EV controls. Notably, SOD2 overexpression significantly potentiated GUTK-induced apoptosis during reactivation in both LNCaP (Figure 3C) and DU145 (Figure 3D) cells. GUTK treatment induced ΔΨm loss in 32.19% of LNCaP EV cells, which significantly increased to 75.72% in SOD2 OE cells during awakening from quiescence (Figure 3E). Similarly, in DU145 cells, GUTK-induced ΔΨm loss increased from 47.51% in EV controls to 95.95% in SOD2 OE cells (Figure 3F). Collectively, these data demonstrate that SOD2 overexpression enhances the pro-apoptotic effects of GUTK, underscoring SOD2 as a pivotal regulator in eliminating reactivating PCa cells.

### GUTK inhibits the recovery of Aurora A and stabilizes SOD2 protein during the reactivation of PCa cells

**Figure 4:**
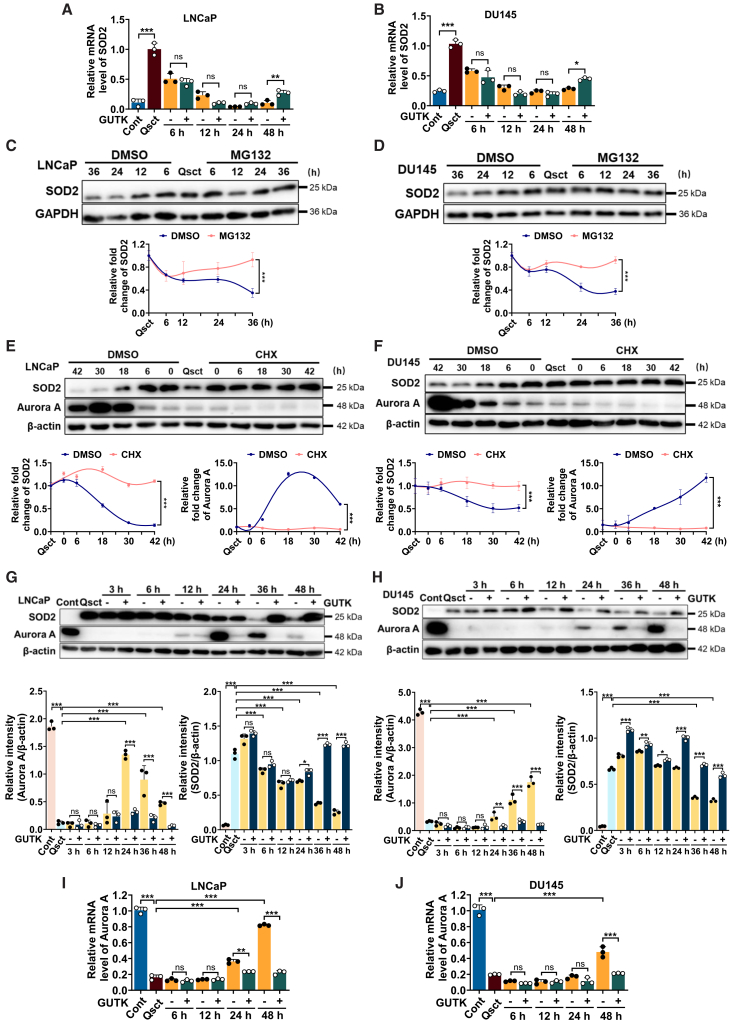
GUTK downregulates Aurora A expression during the reactivation of quiescent PCa cells (A and B) RT-PCR analysis of SOD2 mRNA levels in quiescent LNCaP (A) and DU145 (B) cells treated with or without 20 μM GUTK for the indicated times during cell cycle re-entry. (C and D) Quiescent LNCaP and DU145 cells were induced to re-enter the cell cycle and simultaneously exposed to 10 μM MG132 or DMSO for the indicated times. Immunoblot analysis of SOD2 protein was performed in quiescent LNCaP (C) and DU145 (D) cells. GAPDH served as the loading control. Quantification of relative protein levels is shown in the lower panel. (E and F) Quiescent LNCaP and DU145 cells were induced to re-enter the cell cycle for 6 h and then exposed to 50 μM CHX or DMSO for the indicated times. Immunoblot analysis of SOD2 and Aurora A proteins was performed in quiescent LNCaP (E) and DU145 (F) cells. β-actin or GAPDH served as loading control. Quantification of relative protein levels is shown in the lower panel. (G and H) Immunoblot analysis of Aurora A protein levels in quiescent LNCaP (G) and DU145 (H) cells treated with 20 μM GUTK for the indicated times during cell cycle re-entry. β-actin served as loading control. Quantification of relative protein levels is shown in the lower panel. (I and J) RT-PCR analysis of Aurora A mRNA levels in quiescent LNCaP (I) and DU145 (J) cells treated with 20 μM GUTK for the indicated times during cell cycle re-entry. Cont: proliferating control cells; Qsct: quiescent cells; CHX: cycloheximide. Data are presented as mean ± SD from three independent experiments. ∗*p* < 0.05, ∗∗*p* < 0.01, and ∗∗∗*p* < 0.001 versus indicated group. ns: not significant.

To explore the mechanism by which GUTK sustains elevated SOD2 protein levels, we first examined whether this regulation occurs at the transcriptional level. RT-PCR analysis revealed that SOD2 mRNA levels were significantly higher in quiescent LNCaP and DU145 cells compared to their proliferative counterparts, and gradually declined during cell cycle re-entry (Figures 4A and 4B). However, GUTK treatment showed no significant effect on SOD2 mRNA levels (Figures 4A and 4B), suggesting that GUTK-mediated SOD2 upregulation may not be mediated through transcriptional regulation. Given the established role of the ubiquitin-proteasome pathway in mediating SOD2 degradation,^22^^,^^23^ we treated reactivating PCa cells with the proteasome inhibitor MG132 to assess its effect on SOD2 protein dynamics during reactivation. Immunoblot analysis revealed that, unlike the progressive reduction of SOD2 expression observed in control cells, MG132 treatment effectively prevented SOD2 downregulation during reactivation (Figures 4C and 4D). These results indicate that SOD2 protein levels appear to be regulated by proteasomal degradation during the reactivation process. To further examine SOD2 protein turnover, we used CHX to block protein synthesis during quiescent cell reactivation. Surprisingly, although SOD2 protein levels typically decline during reactivation, CHX treatment unexpectedly preserved SOD2 protein levels (Figures 4E and 4F). This paradoxical stabilization suggests the involvement of a short-lived regulatory protein that promotes SOD2 degradation, which is likely depleted upon CHX treatment.

Aurora A, a predicted E3 ligase for SOD2, has been reported as a potential mediator of SOD2 degradation.^19^ As expected, immunoblot analysis revealed that Aurora A protein levels were low in quiescent PCa cells and significantly increased during reactivation in both LNCaP and DU145 cells. However, this reactivation-induced increase was significantly suppressed by CHX treatment (Figures 4E and 4F), providing a plausible explanation for the paradoxical stabilization of SOD2 upon protein synthesis inhibition. Notably, GUTK treatment significantly downregulated Aurora A expression at both the protein (Figures 4G and 4H) and mRNA levels (Figures 4I and 4J), with its expression inversely correlated with SOD2 upregulation over time. Collectively, these findings demonstrate that GUTK sustains elevated SOD2 protein levels, probably by suppressing its potential E3 ligase Aurora A at both transcriptional and post-translational levels, thereby attenuating SOD2 degradation.

### Aurora A overexpression suppresses GUTK-induced SOD2 upregulation and apoptosis

**Figure 5:**
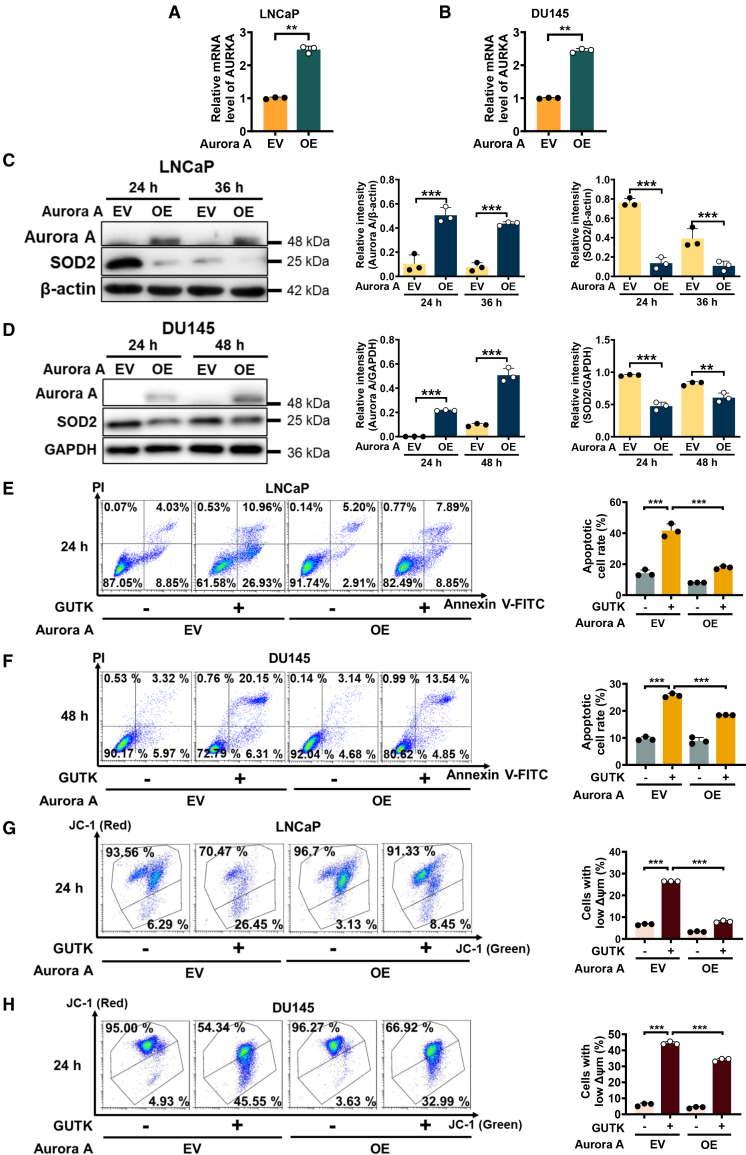
Aurora A overexpression suppresses SOD2 expression and attenuates GUTK-induced apoptosis in reactivating quiescent PCa cells (A and B) RT-PCR validation of Aurora A mRNA overexpression in LNCaP (A) and DU145 (B) cells stably transfected with either empty vector (EV) or an Aurora A expression construct (Aurora A OE). (C and D) Immunoblot analysis of Aurora A and SOD2 protein levels in quiescent EV control or Aurora A OE LNCaP (C) and DU145 (D) cells at the indicated times after cell cycle re-entry. GAPDH served as the loading control. Quantification of relative protein levels is shown in the right panel. (E and F) Apoptosis analysis by Annexin V-FITC/PI staining and flow cytometry in EV control or Aurora A OE LNCaP (E) and DU145 (F) cells treated with or without 20 μM GUTK for the indicated times during cell cycle re-entry. Quantification of apoptotic cell percentages is shown in the right panel. (G and H) ΔΨm analysis by JC-1 staining and flow cytometry in quiescent EV control and Aurora A OE LNCaP (G) and DU145 (H) cells treated with or without 20 μM GUTK for the indicated times during cell cycle re-entry. Quantification of cells with low ΔΨm is shown in the right panel. ΔΨm: mitochondrial membrane potential. Data are presented as mean ± SD from three independent experiments. ∗∗*p* < 0.01 and ∗∗∗*p* < 0.001 versus indicated group.

To determine whether Aurora A is required for GUTK-mediated SOD2 stabilization and apoptosis during quiescent PCa cell reactivation, we established stable PCa cell lines overexpressing Aurora A (Aurora A OE) or EV controls. Successful Aurora A overexpression in LNCaP (Figure 5A) and DU145 (Figure 5B) cells was confirmed by elevated mRNA levels. Consistent with its proposed role in promoting SOD2 degradation, the protein level of SOD2 was markedly reduced in Aurora A OE cells compared to EV controls in both LNCaP (Figure 5C) and DU145 (Figure 5D) cells. Importantly, Aurora A overexpression substantially attenuated GUTK-induced apoptosis, reducing apoptotic rates by approximately 50% and 30% in reactivating quiescent LNCaP (Figure 5E) and DU145 (Figure 5F) cells, respectively. Moreover, Aurora A overexpression alleviated mitochondrial dysfunction, as evidenced by a significant reduction in GUTK-induced ΔΨm loss in both LNCaP (Figure 5G) and DU145 cells (Figure 5H). Collectively, these results demonstrate that the GUTK-induced suppression of Aurora A serves as a key upstream mechanism for SOD2 stabilization and apoptosis induction during the reactivation of quiescent PCa cells.

### Combination of GUTK and Docetaxel inhibits orthotopic prostate tumor growth

**Figure 6:**
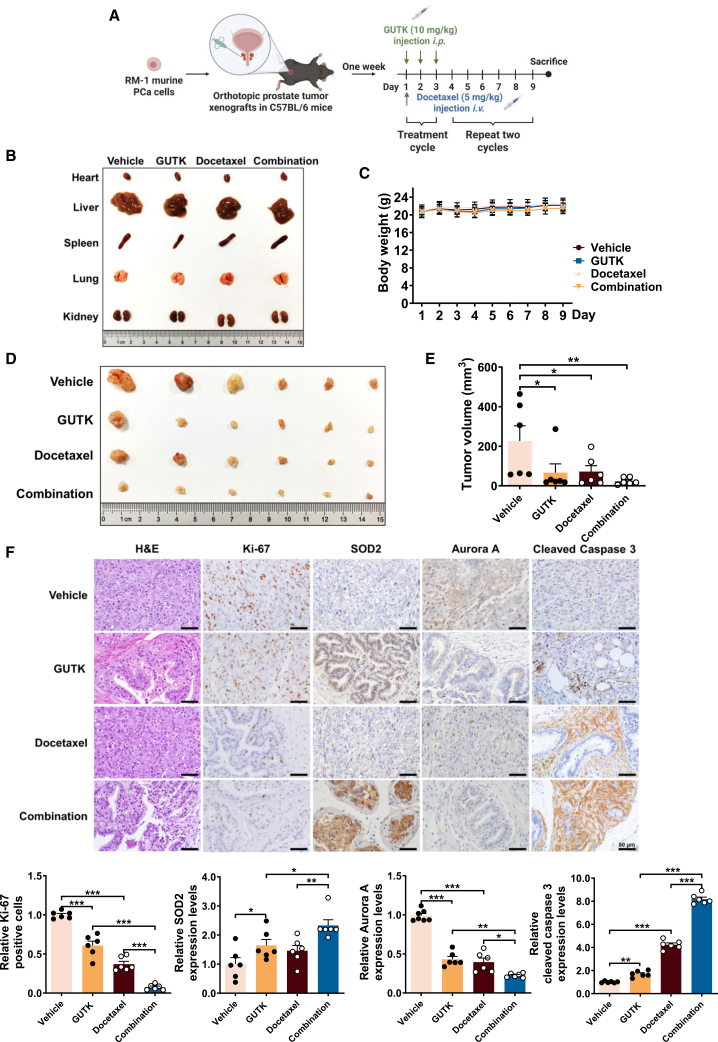
GUTK combined with docetaxel enhances the reduction of orthotopic prostate tumor growth in C57BL/6 mice (A) Experimental schematic of the orthotopic prostate cancer model and treatment schedule in C57BL/6 mice established using RM-1 murine PCa cells. Mice were randomized into four groups: vehicle, GUTK, docetaxel, or combination treatment (*n* = 6 per group). (B) Representative images of major organ morphology (heart, liver, spleen, lungs, kidneys) collected on day 9. (C) Daily body weight measurements throughout the experiment period. (D) Representative images of dissected tumors collected at the day 9 endpoint. (E) Tumor volume quantification at the endpoint. (F) Representative immunohistochemical staining of tumor sections for H&E, Ki-67, SOD2, Aurora A and cleaved caspase 3. Scale bars, 50 μm. Quantification of relative protein levels is shown in the lower panel. Solvent A represents Tween 80: ethanol: saline = 20: 13: 67. Solvent B represents Cremophor EL: Ethanol: 5% glucose = 1: 1: 38. Data are presented as mean ± SEM for each group. ∗*p* < 0.05, ∗∗*p* < 0.01, and ∗∗∗*p* < 0.001 versus vehicle control group.

Given that docetaxel predominantly eliminates proliferating cancer cells but fails to eradicate resistant quiescent populations responsible for recurrence,^24^ combining it with GUTK may potentially improve therapeutic efficacy. To evaluate this combination *in vivo*, we established an orthotopic PCa model by implanting RM-1 cells into the prostatic lobe of C57BL/6 mice. One week after tumor implantation, the mice were randomized into four treatment groups (*n* = 6 per group): vehicle control, docetaxel monotherapy (5 mg/kg), GUTK monotherapy (10 mg/kg), and combination therapy with GUTK and docetaxel (Figure 6A). Treatment-related toxicity was assessed and found to be negligible, as indicated by preserved histological architecture in major organs, including the hearts, livers, spleens, lungs, and kidneys (Figure 6B), and stable body weight throughout the treatment period (Figure 6C). Therapeutic efficacy analysis revealed significant tumor suppression after 9 days of treatment. Both GUTK monotherapy (mean tumor volume: ∼100 mm^3^) and docetaxel monotherapy (mean tumor volume: ∼150 mm^3^) substantially reduced tumor burden compared to vehicle controls (mean tumor volume: ∼400 mm^3^). Notably, the combination therapy group exhibited the most pronounced therapeutic response, with mean tumor volumes reduced to below 20 mm^3^ (Figures 6D and 6E), representing enhanced efficacy compared to either monotherapy.

Histopathological and immunohistochemical profiling of excised tumors provided mechanistic insights (Figure 6F). GUTK-treated tumors exhibited reduced cellular density by H&E staining and significantly decreased Ki-67 positivity, indicating potent anti-proliferative effects. Concurrently, we observed marked upregulation of SOD2, downregulation of Aurora A, and increased expression of cleaved caspase 3.

### GUTK suppresses post-chemotherapy tumor recurrence in a DU145 xenograft model

To further investigate whether GUTK can target cell populations that survive docetaxel treatment, we established a post-chemotherapy tumor recurrence model using subcutaneous DU145 xenografts in nude mice. When tumor volumes reached approximately 400 mm^3^, mice received 15 mg/kg docetaxel *via* tail vein injection every 7 days until the volume regressed to 200 mm^3^, mimicking a primary chemotherapy response. Subsequently, these post-docetaxel mice were randomized into two groups (*n* = 6/group) to receive either daily intraperitoneal injections of 10 mg/kg GUTK or a corresponding vehicle control. The recurrence endpoint was defined as the tumor reaching a volume of 500 mm^3^ or completing 40 days of treatment.

**Figure 7:**
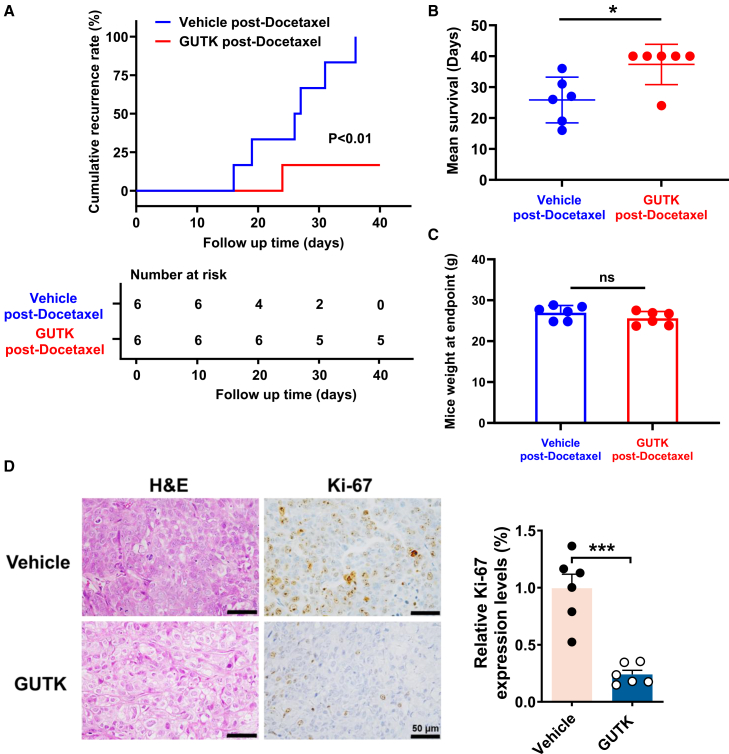
GUTK prolongs post-docetaxel survival in nude mice model (A) Kaplan-Meier analysis of cumulative tumor recurrence in mice treated with docetaxel followed by vehicle or GUTK. The number of animals at risk throughout follow-up is shown below the plot. (B) Quantification of survival time for individual mice in the vehicle and GUTK post-docetaxel groups. Each point represents one mouse, and horizontal bars denote group means. (C) Endpoint body weight measurements of mice in each treatment group. (D) Representative histological images of tumor sections stained with H&E and Ki-67 from vehicle and GUTK post-docetaxel groups. Quantification of relative Ki-67 expression is shown on the right. Scale bars, 50 μm. Data are presented as mean ± SEM for each group. ∗*p* < 0.05 and ∗∗∗*p* < 0.001 versus vehicle control group. ns: not significant.

The results demonstrated that 6 of 6 mice in the vehicle-treated group experienced relapse within 40 days. In contrast, only one mouse in the GUTK-treated group relapsed during the same follow-up period (Figure 7A). GUTK treatment significantly prolonged median survival from 26 days in the vehicle group to 40 days (Figure 7B). Furthermore, no significant difference in body weight was observed between the two groups at the endpoint, indicating that prolonged GUTK administration was well-tolerated (Figure 7C). GUTK-treated post-docetaxel tumors exhibited reduced cellular density by H&E staining and significantly decreased Ki-67 positivity, indicating potent anti-proliferative effects (Figure 7D). Collectively, these findings provide additional evidence that GUTK effectively inhibits tumor recurrence in a post-chemotherapy setting.

## Discussion

Despite significant advances in oncology that have expanded the arsenal of therapeutic modalities available for tumor treatment, cancer remains a formidable global health burden with persistently high mortality rates, largely attributable to its recalcitrant recurrence. This enduring challenge stems primarily from the inability of any single existing modality to achieve the complete eradication of all malignant cells. Residual tumor cells, particularly those residing in a quiescent state, constitute a critical reservoir for disease relapse and metastatic progression. Accordingly, research efforts have increasingly focused on targeting the elusive quiescent tumor cell population as a promising strategy to overcome therapeutic resistance and preventing recurrence.^6^^,^^25^^,^^26^ Current strategies targeting quiescent cancer cells focus on three paradigms: (1) inducing activation to sensitize cells to conventional therapies, (2) enforcing sustained dormancy to prevent recurrence, and (3) direct elimination of quiescent cells by exploiting unique vulnerabilities.^5^ The present findings further identify a novel strategy whereby GUTK directly targets reactivating quiescent PCa cells for elimination *via* mitochondrial apoptosis. It is crucial to distinguish between directly killing quiescent cancer cells and our strategy of eliminating quiescent cancer cells during reactivation. The former aims to target static cells in their deep dormancy, which often proves challenging due to their intrinsic resistance. In contrast, the latter exploits a critical transitional vulnerability, a transient “window of opportunity,” as cells exit quiescence and re-enter the cell cycle, making them susceptible to apoptosis. This makes our strategy potentially broadly applicable against residual disease.

AR signaling remains a central driver of PCa progression and therapeutic response across distinct cellular states. Our exploratory experiments showed that the pharmacological inhibition of AR signaling effectively suppressed cell viability in both proliferative and reactivating quiescent LNCaP cells, underscoring the continued importance of AR activity in these contexts. Notably, the cytotoxic effect of GUTK appeared to be largely independent of AR inhibition, suggesting that GUTK may act through mechanisms distinct from canonical AR signaling pathways. These observations raise the possibility that GUTK could retain efficacy in PCa cells that have developed resistance to AR-targeted therapies. This represents a potentially important therapeutic direction and warrants further investigation into the activity of GUTK in AR-resistant PCa.

Mechanistically, our findings further support our previous report on the paradoxical role of SOD2 during the reactivation of quiescent PCa cells.^9^ SOD2 typically functions as a protective antioxidant.^27^ For example, activated AR signaling functions as a survival mechanism by upregulating antioxidant enzymes, including SOD2, thereby protecting cells from oxidative stress.^28^ However, our data suggest that SOD2 acts as a pro-death effector during reactivation by triggering mitochondrial apoptosis. This conclusion was supported by a significant reduction in GUTK-induced apoptosis following SOD2 knockdown in reactivating quiescent PCa cells. We also explored how GUTK upregulates SOD2 protein levels through transcriptional and post-translational mechanisms. SOD2 transcription is commonly regulated by factors, such as NF-κB, p53, and CCAAT/enhancer-binding protein (C/EBP), through direct DNA binding or protein-protein interactions.^27^ However, RT-PCR analysis showed that GUTK does not alter SOD2 mRNA levels significantly, excluding transcriptional regulation. Instead, MG132 treatment confirmed that SOD2 is regulated through ubiquitin-proteasome-mediated degradation. USP36, a deubiquitinating enzyme known to stabilize nucleolar proteins by reducing their ubiquitination and preventing proteasomal degradation, has been reported to regulate SOD2 stability.^29^ However, our preliminary data showed that GUTK decreases USP36 protein expression (data not shown), which appears contradictory to the observed upregulation of SOD2. This discrepancy suggests the involvement of alternative mechanisms or regulators in SOD2 stabilization under GUTK treatment.

Aurora A is another predicted E3 ligase for SOD2 that has been reported to regulate the degradation of SOD2.^19^ Aurora A is canonically linked to mitotic progression^30^ and chemoresistance in proliferating cells.^31^ However, its role in quiescent cancer cell biology remains unexplored. Our CHX chase experiments suggest that SOD2 degradation during cell cycle re-entry requires continuous synthesis of the short-lived Aurora A protein, which explains why protein synthesis inhibition stabilizes SOD2. Crucially, GUTK downregulates Aurora A at the protein level, leading to SOD2 accumulation. Rescue experiments using Aurora A overexpression confirmed that its downregulation is essential for GUTK-induced apoptosis. This mechanism is distinct from GUTK’s FBXW7/c-MYC-mediated dormancy maintenance pathway, highlighting its multifunctional targeting capacity.^32^

Critically, GUTK addresses an unmet need by eradicating relapse-driving quiescent cancer cells spared by standard chemotherapies such as docetaxel, which primarily target proliferating cancer cells.^33^^,^^34^^,^^35^ Consistent with this notion, GUTK combines with docetaxel to suppress orthotopic PCa tumors *in vivo*. More importantly, the results from our post-chemotherapy recurrence model provide evidence for a compartment-specific targeting hypothesis: Docetaxel eliminates proliferating cancer cells while GUTK targets reactivating quiescent cancer cells. However, elucidating the mechanisms in this experimental design requires more advanced models that recapitulate resident cell behavior post-chemotherapy or in late-stage cancer recurrence. Alternatively, definitive verification of this mechanism requires advanced technologies that are capable of dynamically and accurately distinguishing cellular states. For example, emerging real-time visualization tools (e.g., fluorescent membrane dyes and cell-cycle reporters) will be critical for resolving the spatiotemporal dynamics of GUTK’s activity and advancing its therapeutic development.^36^^,^^37^^,^^38^

In conclusion, this study uncovers a novel mechanism by which GUTK eliminates therapy-resistant quiescent PCa cells during reactivation. We propose a model in which Aurora A-mediated SOD2 degradation preserves mitochondrial homeostasis during reactivation. GUTK disrupts this equilibrium by suppressing Aurora A, leading to SOD2 accumulation and apoptosis in reactivating cells. These findings demonstrate GUTK as a promising therapeutic agent for preventing recurrence and highlight the Aurora A-SOD2 pathway as a potential target for eradicating quiescent cancer cells.

### Limitations of the study

This study has several key limitations that require further investigation: (1) The mechanism underlying SOD2 upregulation and its subsequent activation of apoptotic pathways remains unidentified. It is also unknown whether SOD2 upregulation modulates mitochondrial-related signaling or whether SOD2 expression regulation influences oxidative stress and disrupts redox homeostasis. Future studies should examine whether SOD2 accumulation impairs oxidative phosphorylation^39^^,^^40^ and fatty acid oxidation^41^ pathways, which are essential for the viability of quiescent cancer cells and closely linked to redox homeostasis. (2) Further research is needed to dissect the role of Aurora A’s E3 ligase function in GUTK-induced SOD2 accumulation, specifically investigating how GUTK impairs this activity and mapping the requisite binding sites. Notably, the finding of low Aurora A protein expression in quiescent PCa cells and its gradual recovery during reactivation constitutes a significant observation. This dynamic pattern implies a novel, Aurora A-dependent mechanism regulating quiescent cell reactivation. Future mechanistic studies should prioritize the exploration of this pathway. (3) Moreover, GUTK-mediated transcriptional repression of Aurora A suggests the potential involvement of stress-responsive transcription factors (e.g., FOXM1 or HIF-1α),^42^^,^^43^ representing another avenue for future investigation.

## Resource availability

### Lead contact

Requests for further information and resources should be directed to and will be fulfilled by the lead contact, Prof. Hongxi Xu, (hxxu@shutcm.edu.cn).

### Materials availability

This study did not generate new unique reagents.

### Data and code availability

• Raw data are available from the corresponding author upon reasonable request.
• This paper does not report original code.
• Any additional information required to reanalyze the data reported in this paper is available from the lead contact upon request.

## Acknowledgments

This research was supported by the 10.13039/501100001809National Natural Science Foundation of China (nos. 82204437 and 82404919), the China National Postdoctoral Program for Innovative Talents (no. BX20240230), the Postdoctoral Fellowship Program of 10.13039/501100002858China Postdoctoral Science Foundation (no. GZC20231703, China), the Traditional Chinese Medicine Research Project of the 10.13039/100017950Shanghai Municipal Health Commission (no. 2024QN029), the Shanghai Science and Technology Innovation Action Plan Rising Star Program (no. 24YF2746900), and the Discipline Construction of Pudong Health Bureau of Shanghai-Discipline group of cerebrovascular system diseases (grant no. PWZxq2022-01).

## Author contributions

Y.L.W. and Y.L. contributed to investigation, validation, and data curation. R.C.D. contributed to writing – original draft preparation and editing. Z.C.X. and H.X.X. conceived the research idea and designed the experimental protocols. X.J. and M.F.L. helped the establishment of stable knockdown and overexpression PCa cell lines. H.G.R. and Y.L.Z. provided critical feedback and helped shape the manuscript. X.Q.C., Y.L.W., and R.C.D. helped with the animal experiments. Z.C.X. and H.X.X. supervised the manuscript. R.C.D., Y.L., M.F.L., and H.G.R. contributed to funding acquisition. All authors read and approved the final manuscript.

## Declaration of interests

The authors have declared that no competing interest exists.

## STAR★Methods

### Key resources table

**Table:** 

REAGENT or RESOURCE	SOURCE	IDENTIFIER
**Antibodies**		

PARP Antibody	Cell Signaling Technology	Cat#9542
Caspase-9 Antibody	Cell Signaling Technology	Cat#9502
Caspase-3 Antibody	Cell Signaling Technology	Cat#9662
Superoxide Dismutase 2(E-10)	Santa Cruz	Cat#sc-137254
Aurora A/AIK (1G4) Rabbit Monoclonal Antibody	Cell Signaling Technology	Cat#4718
Androgen Receptor (D6F11) Rabbit Monoclonal Antibody	Cell Signaling Technology	Cat#5153
Beta Actin Monoclonal antibody	Proteintech	Cat#66009-1-Ig
GAPDH Monoclonal antibody	Proteintech	Cat#60004-1-Ig
Anti-rabbit IgG, HRP-linked Antibody	Cell Signaling Technology	Cat#7074
Anti-mouse IgG, HRP-linked Antibody	Cell Signaling Technology	Cat#7076
Anti-Ki-67 (rabbit monoclonal)	Abcam	Cat#ab16667
Anti-SOD2 (rabbit monoclonal, for IHC)	HUABIO	Cat# ET1701-54
Anti-Aurora A (rabbit monoclonal, for IHC)	HUABIO	Cat# ET1609-22
Anti-Cleaved Caspase 3 (rabbit monoclonal, for IHC)	Cell Signaling Technology	Cat# 9661

**Chemicals, peptides, and recombinant proteins**		

Guttiferone K (GUTK)	Isolated from *Garcinia yunnanensis* Hu^44^	N/A
Dimethyl sulfoxide (DMSO)	Sigma-Aldrich	Cat# 543900
Z-VAD-FMK	Beyotime	Cat# C1202
Metribolone (R1881)	GLPBIO	Cat# GC19800
Cycloheximide (CHX)	Sigma-Aldrich	Cat# C7698
MG132	MedChemExpress	Cat# HY-13259
Docetaxel	TargetMol	Cat# T1034
Enzalutamide	TargetMol	Cat# T6002
Apalutamide	TargetMol	Cat# T2339
Puromycin	GeneChem	Cat# REVG1001

**Critical commercial assays**		

Cell Counting Kit-8 (CCK-8)	Beyotime	Cat# C0038
JC-1 Mitochondrial Membrane Potential Assay Kit	Beyotime	Cat# C2006
Annexin V-FITC/PI Apoptosis Detection Kit	Meilun Biotechnology	Cat# MA0220
Annexin V-APC/PI Apoptosis Kit	Multi Sciences Biotech	Cat# AP107
RNAiso Plus (TRIzol)	Takara Biotechnology	Cat# 9108
PrimeScript RT Reagent Kit	Takara Biotechnology	Cat# RR037A
SYBR Green Real-time PCR Master Mix	Toyobo Life Science	Cat# QPK-201

**Experimental models: Cell lines**		

LNCaP	American Type Culture Collection	Cat# CRL-1740; RRID: CVCL_0395
DU145	American Type Culture Collection	Cat# HTB-81; RRID: CVCL_0105
RM-1	American Type Culture Collection	Cat# CRL-3310; RRID: CVCL_B459
HEK-293T	American Type Culture Collection	Cat# CRL-3216; RRID: CVCL_0063

**Experimental models: Organisms/strains**		

Mouse: C57BL/6 (male, 4 weeks old)	Experimental Animal Center of Chinese Academy of Sciences	N/A
Mouse: BALB/c nude (male, 4-6 weeks old)	Experimental Animal Center of Chinese Academy of Sciences	N/A

**Oligonucleotides**		

Primers for SOD2 (forward: ATTTGTAAGTGTCCCCGTTCC; reverse: GTGGTGGTCATATCAATCATAGC)	This paper	N/A
Primers for AURKA (forward: GAGCATCAGCTCAGAAGAGAAG; reverse: GACTCTGGTAGCATCATGGAAATA)	This paper	N/A
Primers for TBP (forward: GAACCACGGCACTGATTTTC; reverse: CCCCACCATGTTCTGAATCT)	This paper	N/A

**Software and algorithms**		

GraphPad Prism 8	GraphPad Software	https://www.graphpad.com/; RRID: SCR_002798
FlowJo v10	FlowJo, LLC	https://www.flowjo.com/; RRID: SCR_008520
ImageJ	NIH	https://imagej.nih.gov/ij/; RRID: SCR_003070
StepOne Software version 2.3	Applied Biosystems	http://downloads.thermofisher.com/Instrument_Software/qPCR/Step-1/SOP23_Release%20Notes_4482516.pdf RRID:SCR_014281

**Other**		

Lenti-Pac HIV Expression Packaging Kit	GeneChem	Cat# GMeasy-10
Polybrene	Santa Cruz	Cat# sc-134220

### Experimental model and study participant details

#### Cell lines

Human PCa cell lines LNCaP (ATCC, CRL-1740) and DU145 (ATCC, HTB-81), human embryonic kidney (HEK)-293T cells (ATCC, CRL-3216) and RM-1 murine PCa cells (ATCC, CRL-3310) were obtained from the American Type Culture Collection (ATCC, Rockville, USA). All cells were cultured at 37 °C in a humidified atmosphere containing 5% CO_2_. PCa cells were maintained in RPMI 1640 medium (MB4374, Meilun Biotechnology, Dalian, China) supplemented with 10% fetal bovine serum (FBS, 04-001-1 ACS, Biological Industries, Beijing, China). HEK-293T cells were cultured in DMEM (MA0212, Meilun Biotechnology, Dalian, China) supplemented with 10% FBS. All culture supplies were obtained from NEST Biotechnology, Wuxi, China. The sex of LNCaP and DU145 cells is male (derived from human male prostate); RM-1 cells are male (derived from C57BL/6 mouse prostate); HEK-293T cells are female (derived from human female embryonic kidney). All cell lines were authenticated by short tandem repeat (STR) profiling and tested negative for mycoplasma contamination using PCR-based methods.

#### Animals

Male C57BL/6 mice (4 weeks old) and male BALB/c nude mice (4-6 weeks old) were obtained from the Experimental Animal Center of the Chinese Academy of Sciences (Shanghai) and maintained under specific pathogen-free (SPF) conditions with controlled temperature, humidity, and a 12-h light/dark cycle. All animal procedures were approved by the Animal Ethics Committee of Shanghai University of Traditional Chinese Medicine (Approval NO. PZSHUTCM2501170002) and conducted in accordance with institutional guidelines.

For orthotopic prostate tumor model, C57BL/6 mice were injected with RM-1 cells into the prostate lobe as described in method details. For post-chemotherapy recurrence model, BALB/c nude mice were injected subcutaneously with DU145 cells as described in method details. No statistical method was used to predetermine sample size; sample sizes were chosen based on prior experience with these models to ensure adequate statistical power. Tumor-bearing mice were randomly assigned to treatment groups. Investigators were not blinded to group allocation during experiments and outcome assessment.

### Method details

#### Chemicals and reagents

GUTK (purity > 98%, isolated from *Garcinia yunnanensis* Hu^44^ was dissolved in dimethyl sulfoxide (DMSO, 543900, Sigma-Aldrich, St. Louis, USA) and stored at -80 °C. Cell Counting Kit-8 (CCK-8, C0038), Z-VAD-FMK (Z-VAD, C1202), RIPA lysis buffer (P0013C) and the Mitochondrial Membrane Potential Assay Kit with JC-1 (C2006) were purchased from Beyotime Institute of Biotechnology (Shanghai, China). Metribolone (R1881, GC19800) was purchased from GLPBIO (California, USA). RNAiso Plus (TRIzol) and PrimeScript RT Reagent Kit (RR037A) were obtained from Takara Biotechnology (Kusatsu, Japan). The Annexin V-FITC/PI Apoptosis Detection Kit was purchased from Meilun Biotechnology (Dalian, China), and the Annexin V-APC/PI Apoptosis Kit from Multi Sciences Biotech (Hangzhou, China). Cycloheximide (CHX, 50 μM, C7698) was obtained from Sigma-Aldrich (St. Louis, USA). MG132 (HY-13259, 10 μM) was obtained from MedChemExpress (New Jersey, USA). Docetaxel (T1034), Enzalutamide (T6002) and Apalutamide (T2339) were purchased from TargetMol (Boston, USA). Puromycin (REVG1001) and lentiviral packaging reagents (Lenti-PacTM HIV Expression Packaging Kit) were from GeneChem (Shanghai, China).

#### Cell lines and culture

Human PCa cell lines LNCaP and DU145, human embryonic kidney (HEK)-293T cells and RM-1 murine PCa cells (American Type Culture Collection, Rockville, USA), were cultured as described in experimental model and study participant details. To induce quiescence, subconfluent (70-80%) LNCaP and DU145 cells were cultured in serum-free RPMI 1640 medium for 7 days. Quiescent cancer cells were trypsinized, replated at the indicated densities in complete medium (RPMI 1640 with 10% FBS), and allowed to re-enter the cell cycle for the specified durations, as previously described.^32^

#### Establishment of doxycycline (DOX)-inducible stable SOD2 shRNA cell lines

Lentiviral vectors encoding DOX-inducible shRNA targeting SOD2 (shSOD2) or a non-targeting control were packaged in HEK-293T cells using the Lenti-Pac^TM^ HIV Expression Packaging Kit (GeneCopoeia, Changzhou, China). Viral supernatants were collected 48-72 h post-transfection, filtered through a 0.45 μm membrane, and used to infect LNCaP and DU145 cells in the presence of 8 μg/mL polybrene. Stable cell lines were selected with 0.2 mg/mL puromycin. SOD2 knockdown was induced by adding 1 μg/mL DOX 48 h prior to experimentation.

#### Establishment of stable PCa cell lines overexpressing SOD2 or Aurora A

LNCaP and DU145 cells were infected with lentivirus carrying human SOD2 cDNA (Ubi-MCS-3FLAG-SV40-puromycin-SOD2, GenoChem, Shanghai, China) or AURKA cDNA (Ubi-MCS-3FLAG-SV40-puromycin-AURKA, GenoChem, Shanghai, China), respectively. Cells transduced with the empty vector (EV, Ubi-MCS-3FLAG-SV40-puromycin, GenoChem, Shanghai, China) serves as controls. Stable pools were selected using 0.2 mg/mL puromycin. Overexpression of SOD2 and Aurora A was confirmed by RT-PCR or immunoblot analysis.

#### Cell viability assay

During reactivation, quiescent LNCaP (1 × 10^5^ cells/well) or DU145 cells (8 × 10^4^ cells/well) were seeded in 96-well plates in complete medium containing vehicle control, the indicated concentrations of GUTK, R1881, Docetaxel, enzalutamide or apalutamide. After 48 h of incubation, cell viability was assessed by using the CCK-8 according to the manufacturer’s instructions (Meilun Biotechnology, Dalian, China). Absorbance was measured at 450 nm using a microplate reader (PerkinElmer, Shanghai, China).

#### Immunoblot analysis

Cell pellets were lysed in ice-cold RIPA buffer supplemented with a protease inhibitor cocktail. Protein quantification, electrophoresis, transfer and immunoblot analysis were performed as previously described.^9^ Membranes were blocked for nonspecific binding with 10% skim milk powder in Tris Buffered Saline with Tween (TBST) and subsequently incubated overnight at 4 °C with the following primary antibodies: anti-SOD2 (sc-137254) from Santa Cruz Biotechnology (Dallas, USA); anti-Caspase 3 (9662), anti-poly ADP-ribose polymerase (PARP, 9542), anti-Caspase 9 (9502) and Aurora A (4718s) from Cell Signaling Technology (Danvers, USA); β-actin (66009-1-Ig) and GAPDH (60004-1-Ig) from Proteintech (Wuhan, China). Membranes were subsequently probed with horseradish peroxidase (HRP)-conjugated goat anti-rabbit or goat anti-mouse secondary antibodies (1:10,000; Santa Cruz Biotechnology, Dallas, USA), and protein bands were visualized using Chemiluminescence imaging system (Bio-Rad, Hercules, USA).

#### Mitochondrial membrane potential (MMP, ΔΨm) analysis

MMP was assessed using the JC-1 staining kit (C2006, Beyotime Institute of Biotechnology, Shanghai, China) during cell cycle re-entry as previously described.^45^ Quiescent PCa cells were seeded in 6-cm dishes (1 × 10^6^ cells per dish) in complete medium containing DMSO (vehicle control) or 20 μM GUTK. After 24 h, 36 h or 48 h of incubation, cells were harvested, washed with PBS, and stained with JC-1 working solution (5 μg/mL) at 37 °C for 20 min. Cells were then washed twice with JC-1 washing buffer, resuspended in assay buffer, and immediately analyzed by flow cytometry (FACS Calibur, BD Biosciences, San Jose, USA). JC-1 aggregates (indicative of high ΔΨm, red fluorescence, FL2 channel ∼ 585 nm) and monomers (indicative of low ΔΨm, green fluorescence, FL1 channel ∼ 530 nm) were detected. The percentage of cells exhibiting low ΔΨm was quantified using FlowJo software. A minimum of 10,000 events were acquired per sample.

#### Annexin V/PI double staining assay

Quiescent PCa cells were plated in 6-cm dishes (1 × 10^6^ cells per dish) in complete medium containing DMSO, 20 μM GUTK, 50 μM Z-VAD-FMK, or 20 μM GUTK plus 50 μM Z-VAD-FMK. After incubation for 24 h, 36 h or 48 h, cells were harvested, washed twice with ice-cold PBS, and stained using either the Annexin V-FITC/PI Kit or the Annexin V-APC/PI Kit (used specifically for LNCaP shSOD2 and DU145 shSOD2 cells) according to the manufacturer’s instructions. Stained cells were analyzed by flow cytometry (FACS Calibur, BD Biosciences, San Jose, USA) within 1 h. Annexin V-positive cells were quantified as the apoptotic population. A minimum of 10,000 events was acquired per sample.

#### Reverse transcription-polymerase chain reaction (RT-PCR)

Total RNA was extracted from cells using TRIzol reagent, and its concentration and purity were determined using a NanoDrop spectrophotometer (DeNovix, Wilmington, USA). cDNA was synthesized from 2 μg total RNA using the PrimeScript RT Reagent Kit (Takara Biotechnology, Kusatsu, Japan). RT-PCR was performed using SYBR Green Real-time PCR Master Mix on a StepOnePlus System (Applied Biosystems, Carlsbad, USA). Each 10 μL reaction contained 1 μL cDNA, 5 μL SYBR Green Mix 3.5 μL Diethylpyrocarbonate water and 0.5 μL of each primer. The thermal cycling conditions were: 95 °C for 30 sec, followed by 40 cycles of 95 °C for 5 sec and 60 °C for 30 sec. Melt curve analysis was conducted to confirm amplification specificity. TATA box-binding protein (TBP) was used as the endogenous control. The primer sequences were as follows: 5′-GAAC CACG GCAC TGAT TTTC-3′ (forward) and 5′-CCCC ACCA TGTT CTGA ATCT-3′ (reverse) for TBP; 5′-ATTT GTAA GTGT CCCC GTTC C-3′ (forward) and 5′-GTGG TGGT CATA TCAA TCAT AGC-3′ (reverse) for SOD2; 5′-GAGC ATCA GCTC AGAA GAGA AG-3′ (forward) and 5′- GACT CTGG TAGC ATCA TGGA AATA-3′ (reverse) for AURKA. Gene expression levels were quantified using StepOne Software version 2.3 and analyzed with Microsoft Excel.

#### Orthotopic prostate tumor xenografts in C57BL/6 mice

Male C57BL/6 mice (4 weeks old) were used as described in experimental model and study participant details. RM-1 murine PCa cells were prepared by trypsinization, and cell viability was confirmed using trypan blue exclusion assay. Viable cells were suspended in PBS at 2.67 × 10^4^ cells/mL and kept on ice prior to injection. Mice were anesthetized with 3% sodium pentobarbital (intraperitoneal injection, *i.p.*), and a lower abdominal incision was made to expose the prostate. A total of 75 μL of the RM-1 cell suspension (∼2.0 × 10^3^ cells) was injected into the prostate lobe. One week after implantation, tumor-bearing mice were randomized into four groups (n = 6 per group): Control, GUTK, Docetaxel and Combination group. Docetaxel was dissolved by solvent A (Tween 80: Ethanol: saline = 20: 13: 67). GUTK was dissolved by solvent B (Cremophor EL: Ethanol: 5% glucose = 1: 1: 38). Specifically, Control group received intravenous (*i.v.*) injection of solvent A every 3 days plus daily *i.p.* injection of solvent B; GUTK group received *i.v.* injection of solvent A every 3 days plus daily *i.p.* injection of GUTK (10 mg/kg); Docetaxel group received *i.v.* injection of Docetaxel (5 mg/kg) every 3 days plus daily *i.p.* injection of solvent B; Combination group received *i.v.* injection of Docetaxel (5 mg/kg) every 3 days plus daily *i.p.* injection of GUTK (10 mg/kg). After three treatment cycles, mice were euthanized. Each cycle consisted of a single dose of Docetaxel on Day 1 and daily administration of GUTK from Day 1 to Day 3 (3 consecutive days per cycle). Prostate tumors were dissected, weighed and measured using digital calipers. Tumor volume was calculated using the formula: volume = (length × width^2^) / 2, with length representing the longest diameter and width the shortest.

#### Establishment of post-chemotherapy recurrence model in subcutaneous DU145 xenograft nude mice

Male BALB/c nude mice (4-6 weeks old) were used as described in experimental model and study participant details. Human prostate cancer DU145 cells were cultured under standard conditions and harvested during the exponential growth phase. A total of 5 × 10^6^ DU145 cells suspended in 100 μL of PBS were injected subcutaneously into the right flank of nude mice. Tumor dimensions were measured with digital calipers every day. When tumor volume reached approximately 400 mm^3^, mice were administered Docetaxel (15 mg/kg) by tail *i.v.* injection every 7 days. Docetaxel treatment continued until tumors regressed to approximately 200 mm^3^ to mimic a primary chemotherapy response. Mice that achieved this post-treatment regression were subsequently randomized into two groups (n = 6 per group): Vehicle control-daily received *i.p.* injection of solvent B; GUTK treatment-daily *i.p.* injection of GUTK (10 mg/kg). Following randomization, mice received treatment for up to 40 days or until tumor recurrence. The recurrence endpoint was defined as tumor regrowth to 500 mm^3^ or completion of the 40-day follow-up period. Mice were monitored for tumor size, general health, and body weight throughout the study. Survival time was recorded from the first day of randomization to the recurrence endpoint.

#### Immunohistochemistry

Following euthanasia, tumors were immediately collected and fixed in 4% paraformaldehyde (PFA). Fixed tissues were paraffin-embedded and sectioned at a thickness of 5 μm. Sections were subjected to hematoxylin and eosin (H&E) staining or immunohistochemical staining using primary antibodies against Ki-67 (ab16667, Abcam, Shanghai, China), SOD2 (ET1701-54, HUABIO, Hangzhou, China), Cleaved Caspase 3 (#9661, Cell Signaling Technology, Danvers, USA) and Aurora A (ET1609-22, HUABIO, Hangzhou, China). Stained sections were mounted with DPX for histological examination. For quantitative analysis, at least three random fields per section (n = 3 per group) were imaged. The positively stained area in each field was quantified using ImageJ software by applying a standardized threshold to isolate specific staining and calculating the percentage of positive area (% area).

### Quantification and statistical analysis

Data are presented as mean ± standard deviation (SD) or standard error of the mean (SEM) from at least three independent experiments. For *in vitro* experiments, n represents the number of independent biological replicates; for *in vivo* studies, n represents the number of animals per group. Tumor recurrence curves were analyzed using Kaplan-Meier survival analysis and log-rank test. Statistical significance was determined using Student's t-test or one-way/two-way analysis of variance (ANOVA) using GraphPad Prism 8.0. If assumptions of normality were not met, non-parametric tests were applied using the Kruskal-Wallis test followed by Mann-Whitney U tests with Holm correction for multiple comparisons. No data were excluded from the analyses. Significance levels are indicated as follows: ∗*P* < 0.05, ∗∗*P* < 0.01, ∗∗∗*P* < 0.001; ns: not significant.

## References

[1] Chang A.J., Autio K.A., Roach M., Scher H.I. (2014). High-risk prostate cancer-classification and therapy. Nat. Rev. Clin. Oncol..

[2] Kishan A.U., Cook R.R., Ciezki J.P., Ross A.E., Pomerantz M.M., Nguyen P.L., Shaikh T., Tran P.T., Sandler K.A., Stock R.G. (2018). Radical Prostatectomy, External Beam Radiotherapy, or External Beam Radiotherapy With Brachytherapy Boost and Disease Progression and Mortality in Patients With Gleason Score 9-10 Prostate Cancer. JAMA.

[3] Yeh A.C., Ramaswamy S. (2015). Mechanisms of Cancer Cell Dormancy--Another Hallmark of Cancer?. Cancer Res..

[4] Tau S., Miller T.W. (2023). The role of cancer cell bioenergetics in dormancy and drug resistance. Cancer Metastasis Rev..

[5] Nik Nabil W.N., Xi Z., Song Z., Jin L., Zhang X.D., Zhou H., De Souza P., Dong Q., Xu H. (2021). Towards a Framework for Better Understanding of Quiescent Cancer Cells. Cells.

[6] Recasens A., Munoz L. (2019). Targeting Cancer Cell Dormancy. Trends Pharmacol. Sci..

[7] Sun Y., Chen Y., Liu Z., Wang J., Bai J., Du R., Long M., Shang Z. (2024). Mitophagy-Mediated Tumor Dormancy Protects Cancer Cells from Chemotherapy. Biomedicines.

[8] Sansone P., Savini C., Kurelac I., Chang Q., Amato L.B., Strillacci A., Stepanova A., Iommarini L., Mastroleo C., Daly L. (2017). Packaging and transfer of mitochondrial DNA via exosomes regulate escape from dormancy in hormonal therapy-resistant breast cancer. Proc. Natl. Acad. Sci. USA.

[9] Xi Z., Liu M., Jiang X., Feng J., Dai R., Nik Nabil W.N., Sun X., Chen J., Ren H., Zhang J. (2025). Pterostilbene Induces Apoptosis in Awakening Quiescent Prostate Cancer Cells by Upregulating C/EBP-β-Mediated SOD2 Transcription. Int. J. Biol. Sci..

[10] Flynn J.M., Melov S. (2013). SOD2 in mitochondrial dysfunction and neurodegeneration. Free Radic. Biol. Med..

[11] Tamagawa S., Sakai D., Nojiri H., Nakamura Y., Warita T., Matsushita E., Schol J., Soma H., Ogasawara S., Munesada D. (2024). SOD2 orchestrates redox homeostasis in intervertebral discs: A novel insight into oxidative stress-mediated degeneration and therapeutic potential. Redox Biol..

[12] Sarsour E.H., Kalen A.L., Xiao Z., Veenstra T.D., Chaudhuri L., Venkataraman S., Reigan P., Buettner G.R., Goswami P.C. (2012). Manganese superoxide dismutase regulates a metabolic switch during the mammalian cell cycle. Cancer Res..

[13] Nikonova A.S., Astsaturov I., Serebriiskii I.G., Dunbrack R.L., Golemis E.A. (2013). Aurora A kinase (AURKA) in normal and pathological cell division. Cell. Mol. Life Sci..

[14] Lim D.C., Joukov V., Rettenmaier T.J., Kumagai A., Dunphy W.G., Wells J.A., Yaffe M.B. (2020). Redox priming promotes Aurora A activation during mitosis. Sci. Signal..

[15] Guarino Almeida E., Renaudin X., Venkitaraman A.R. (2020). A kinase-independent function for AURORA-A in replisome assembly during DNA replication initiation. Nucleic Acids Res..

[16] Naso F.D., Boi D., Ascanelli C., Pamfil G., Lindon C., Paiardini A., Guarguaglini G. (2021). Nuclear localisation of Aurora-A: its regulation and significance for Aurora-A functions in cancer. Oncogene.

[17] Sun H., Wang H., Wang X., Aoki Y., Wang X., Yang Y., Cheng X., Wang Z., Wang X. (2020). Aurora-A/SOX8/FOXK1 signaling axis promotes chemoresistance via suppression of cell senescence and induction of glucose metabolism in ovarian cancer organoids and cells. Theranostics.

[18] Mou P.K., Yang E.J., Shi C., Ren G., Tao S., Shim J.S. (2021). Aurora kinase A, a synthetic lethal target for precision cancer medicine. Exp. Mol. Med..

[19] Yang C., You D., Huang J., Yang B., Huang X., Ni J. (2019). Effects of AURKA-mediated degradation of SOD2 on mitochondrial dysfunction and cartilage homeostasis in osteoarthritis. J. Cell. Physiol..

[20] Wang Z., Liu Z., Qu J., Sun Y., Zhou W. (2024). Role of natural products in tumor therapy from basic research and clinical perspectives. Acta Mater. Med..

[21] Nik Nabil W.N., Xi Z., Liu M., Li Y., Yao M., Liu T., Dong Q., Xu H. (2022). Advances in therapeutic agents targeting quiescent cancer cells. Acta Mater. Med..

[22] Schultz A., Olorundami O.A., Teng R.-J., Jarzembowski J., Shi Z.-Z., Kumar S.N., Pritchard K., Konduri G.G., Afolayan A.J. (2019). Decreased OLA1 (Obg-Like ATPase-1) Expression Drives Ubiquitin-Proteasome Pathways to Downregulate Mitochondrial SOD2 (Superoxide Dismutase) in Persistent Pulmonary Hypertension of the Newborn. Hypertension.

[23] Adams B., Mapanga R.F., Essop M.F. (2015). Partial inhibition of the ubiquitin-proteasome system ameliorates cardiac dysfunction following ischemia-reperfusion in the presence of high glucose. Cardiovasc. Diabetol..

[24] Feng J., Xi Z., Jiang X., Li Y., Nik Nabil W.N., Liu M., Song Z., Chen X., Zhou H., Dong Q., Xu H. (2023). Saikosaponin A enhances Docetaxel efficacy by selectively inducing death of dormant prostate cancer cells through excessive autophagy. Cancer Lett..

[25] Phan T.G., Croucher P.I. (2020). The dormant cancer cell life cycle. Nat. Rev. Cancer.

[26] Tufail M., Jiang C.-H., Li N. (2025). Tumor dormancy and relapse: understanding the molecular mechanisms of cancer recurrence. Mil. Med. Res..

[27] Liu M., Sun X., Chen B., Dai R., Xi Z., Xu H. (2022). Insights into Manganese Superoxide Dismutase and Human Diseases. Int. J. Mol. Sci..

[28] Kajihara T., Tochigi H., Prechapanich J., Uchino S., Itakura A., Brosens J.J., Ishihara O. (2012). Androgen signaling in decidualizing human endometrial stromal cells enhances resistance to oxidative stress. Fertil. Steril..

[29] Kim M.-S., Ramakrishna S., Lim K.-H., Kim J.-H., Baek K.-H. (2011). Protein stability of mitochondrial superoxide dismutase SOD2 is regulated by USP36. J. Cell. Biochem..

[30] Glover D.M. (2003). Aurora A on the mitotic spindle is activated by the way it holds its partner. Mol. Cell.

[31] Cammareri P., Scopelliti A., Todaro M., Eterno V., Francescangeli F., Moyer M.P., Agrusa A., Dieli F., Zeuner A., Stassi G. (2010). Aurora-a is essential for the tumorigenic capacity and chemoresistance of colorectal cancer stem cells. Cancer Res..

[32] Xi Z., Yao M., Li Y., Xie C., Holst J., Liu T., Cai S., Lao Y., Tan H., Xu H.X., Dong Q. (2016). Guttiferone K impedes cell cycle re-entry of quiescent prostate cancer cells via stabilization of FBXW7 and subsequent c-MYC degradation. Cell Death Dis..

[33] Llinas-Bertran A., Bellet-Ezquerra M., Seoane J.A. (2024). Epigenetic Control of Cancer Cell Dormancy and Awakening in Endocrine Therapy Resistance. Cancer Discov..

[34] Ma B., Wells A., Wei L., Zheng J. (2021). Prostate cancer liver metastasis: Dormancy and resistance to therapy. Semin. Cancer Biol..

[35] Francescangeli F., De Angelis M.L., Rossi R., Cuccu A., Giuliani A., De Maria R., Zeuner A. (2023). Dormancy, stemness, and therapy resistance: interconnected players in cancer evolution. Cancer Metastasis Rev..

[36] Lawson M.A., McDonald M.M., Kovacic N., Hua Khoo W., Terry R.L., Down J., Kaplan W., Paton-Hough J., Fellows C., Pettitt J.A. (2015). Osteoclasts control reactivation of dormant myeloma cells by remodelling the endosteal niche. Nat. Commun..

[37] Okreglak V., Ling R., Ingaramo M., Thayer N.H., Millett-Sikking A., Gottschling D.E. (2023). Cell cycle-linked vacuolar pH dynamics regulate amino acid homeostasis and cell growth. Nat. Metab..

[38] Herz K., Becker A., Shi C., Ema M., Takahashi S., Potente M., Hesse M., Fleischmann B.K., Wenzel D. (2018). Visualization of endothelial cell cycle dynamics in mouse using the Flt-1/eGFP-anillin system. Angiogenesis.

[39] Lagadinou E.D., Sach A., Callahan K., Rossi R.M., Neering S.J., Minhajuddin M., Ashton J.M., Pei S., Grose V., O'Dwyer K.M. (2013). BCL-2 inhibition targets oxidative phosphorylation and selectively eradicates quiescent human leukemia stem cells. Cell Stem Cell.

[40] Li Y., Chen H., Xie X., Yang B., Wang X., Zhang J., Qiao T., Guan J., Qiu Y., Huang Y.X. (2023). PINK1-Mediated Mitophagy Promotes Oxidative Phosphorylation and Redox Homeostasis to Induce Drug-Tolerant Persister Cancer Cells. Cancer Res..

[41] Kalucka J., Bierhansl L., Conchinha N.V., Missiaen R., Elia I., Brüning U., Scheinok S., Treps L., Cantelmo A.R., Dubois C. (2018). Quiescent Endothelial Cells Upregulate Fatty Acid β-Oxidation for Vasculoprotection via Redox Homeostasis. Cell Metab..

[42] Mancini M., De Santis S., Monaldi C., Bavaro L., Martelli M., Castagnetti F., Gugliotta G., Rosti G., Santucci M.A., Martinelli G. (2019). Hyper-activation of Aurora kinase a-polo-like kinase 1-FOXM1 axis promotes chronic myeloid leukemia resistance to tyrosine kinase inhibitors. J. Exp. Clin. Cancer Res..

[43] Wan X.-B., Fan X.-J., Huang P.-Y., Dong D., Zhang Y., Chen M.-Y., Xiang J., Xu J., Liu L., Zhou W.H. (2012). Aurora-A activation, correlated with hypoxia-inducible factor-1α, promotes radiochemoresistance and predicts poor outcome for nasopharyngeal carcinoma. Cancer Sci..

[44] Xu G., Feng C., Zhou Y., Han Q.-B., Qiao C.-F., Huang S.-X., Chang D.C., Zhao Q.S., Luo K.Q., Xu H.X. (2008). Bioassay and ultraperformance liquid chromatography/mass spectrometry guided isolation of apoptosis-inducing benzophenones and xanthone from the pericarp of Garcinia yunnanensis Hu. J. Agric. Food Chem..

[45] Nie S., Shi Z., Shi M., Li H., Qian X., Peng C., Ding X., Zhang S., Lv Y., Wang L. (2021). PPARγ/SOD2 Protects Against Mitochondrial ROS-Dependent Apoptosis via Inhibiting ATG4D-Mediated Mitophagy to Promote Pancreatic Cancer Proliferation. Front. Cell Dev. Biol..

