# Supplementary material for: GUTK induces apoptosis in reactivating quiescent prostate cancer cells via Aurora A-mediated stabilization of SOD2
Source: iScience. 2026 Apr 15;29(5):115739. doi: 10.1016/j.isci.2026.115739 (PMC13141025; doi:10.1016/j.isci.2026.115739)
Supplement: Document S1. Figure S1 and Data S1 [file mmc1.pdf]

iScience, Volume *29*

Supplemental information

GUTK induces apoptosis in reactivating quiescent prostate cancer cells *via* Aurora A-mediated stabilization of SOD2

Yalin Wang, Yang Li, Xue Jiang, Xiaoqiong Chen, Mengfan Liu, Hangui Ren, Yulong Zhang, Rongchen Dai, Zhichao Xi, and Hongxi Xu

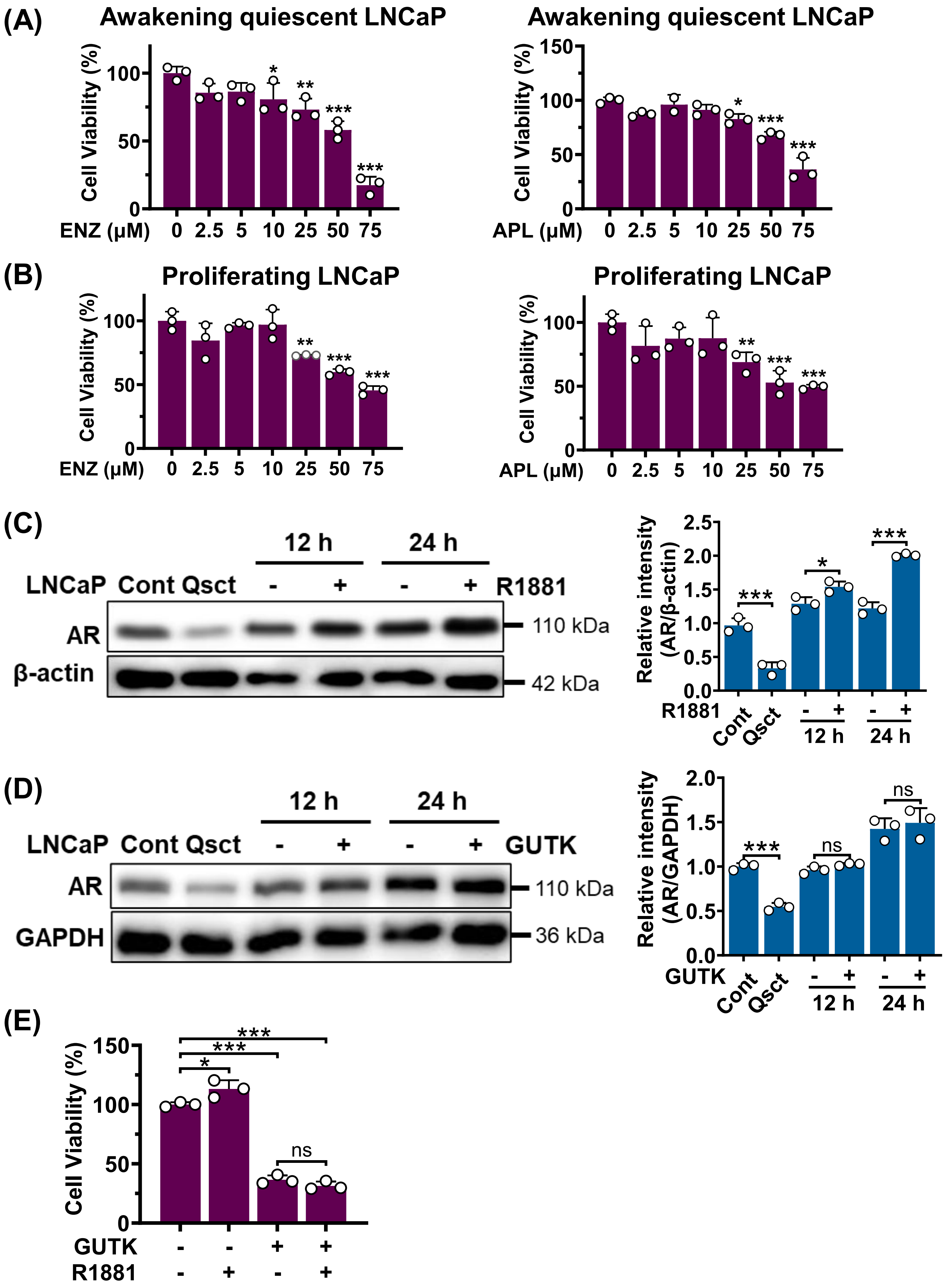
(A)
Awakening quiescent LNCaP
Cell Viability (%)
ENZ (μM)
0
2.5
5
10
25
50
75
*
**
***
***
Awakening quiescent LNCaP
Cell Viability (%)
APL (μM)
*
***
***
(B)
Proliferating LNCaP
Cell Viability (%)
ENZ (μM)
**
***
***
Proliferating LNCaP
Cell Viability (%)
APL (μM)
**
***
***
(C)
12 h
24 h
LNCaP Cont Qsct - + - + R1881
AR
β-actin
110 kDa
42 kDa
Relative intensity (AR/β-actin)
R1881
Cont
Qsct
12 h
24 h
***
*
***
(D)
12 h
24 h
LNCaP Cont Qsct - + - + GUTK
AR
GAPDH
110 kDa
36 kDa
Relative intensity (AR/GAPDH)
GUTK
Cont
Qsct
12 h
24 h
***
ns
ns
(E)
Cell Viability (%)
GUTK - - + +
R1881 - + - +
*
***
***
ns

Supplementary Figure 1. GUTK induces cell death largely independent of androgen receptor signaling during PCa cell reactivation, related to Figure 1.

(A) Cell viability assessed by CCK-8 assay in quiescent LNCaP cells treated with indicated concentration of enzalutamide (ENZ) or apalutamide (APL) for 48 h during reactivation. **(B)** Cell viability assessed by CCK-8 assay in proliferative LNCaP cells treated with indicated concentration of ENZ or APL for 48 h. **(C)** Immunoblot analysis of AR proteins was performed in quiescent LNCaP cells treated with 1 nM R1881 for 24 h during cell cycle re-entry. β-actin served as loading control. Quantification of relative protein levels is shown on the right. **(D)** Immunoblot analysis of AR was performed in quiescent LNCaP cells treated with 20 μM GUTK for 24 h during cell cycle re-entry. GAPDH served as loading control. Quantification of relative protein levels is shown on the right. **(E)** Cell viability assessed by CCK-8 assay in quiescent LNCaP cells treated with 20 μM GUTK, 1 nM R1881 or a combination of both for 48 h during reactivation. Data are presented as mean ± SD from three independent experiments. *$P < 0.05$, **$P < 0.01$ and ***$P < 0.001$ versus indicated group. ns: not significant.

Figure 1E
N=1

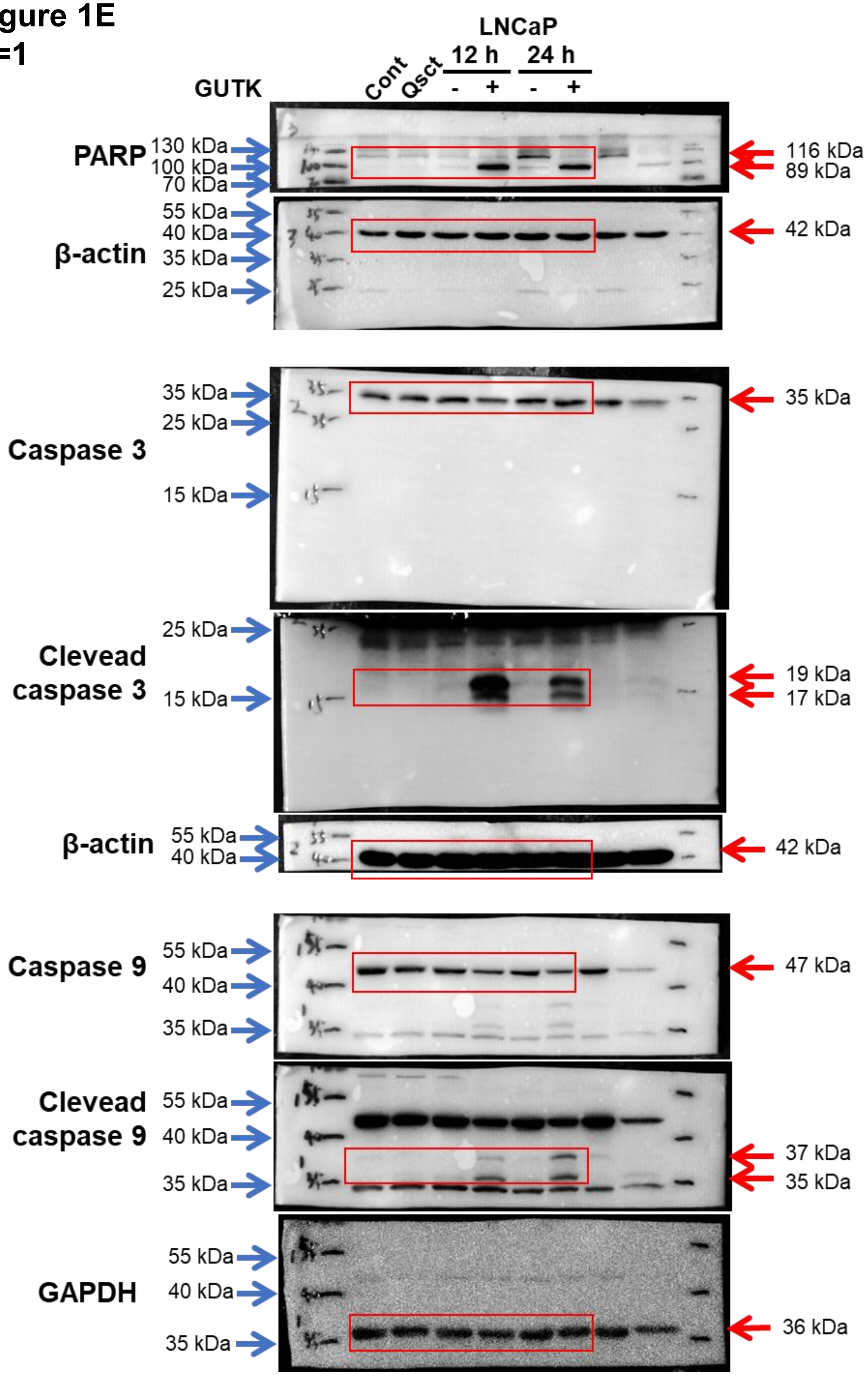

LNCaP
GUTK
Cont
Qsct
12 h
24 h
-
+
-
+
PARP
130 kDa
100 kDa
70 kDa
116 kDa
89 kDa
β-actin
55 kDa
40 kDa
35 kDa
25 kDa
42 kDa
Caspase 3
35 kDa
25 kDa
15 kDa
35 kDa
Clevead caspase 3
25 kDa
15 kDa
19 kDa
17 kDa
β-actin
55 kDa
40 kDa
42 kDa
Caspase 9
55 kDa
40 kDa
35 kDa
47 kDa
Clevead caspase 9
55 kDa
40 kDa
35 kDa
37 kDa
35 kDa
GAPDH
55 kDa
40 kDa
35 kDa
36 kDa

Figure 1E
N=2

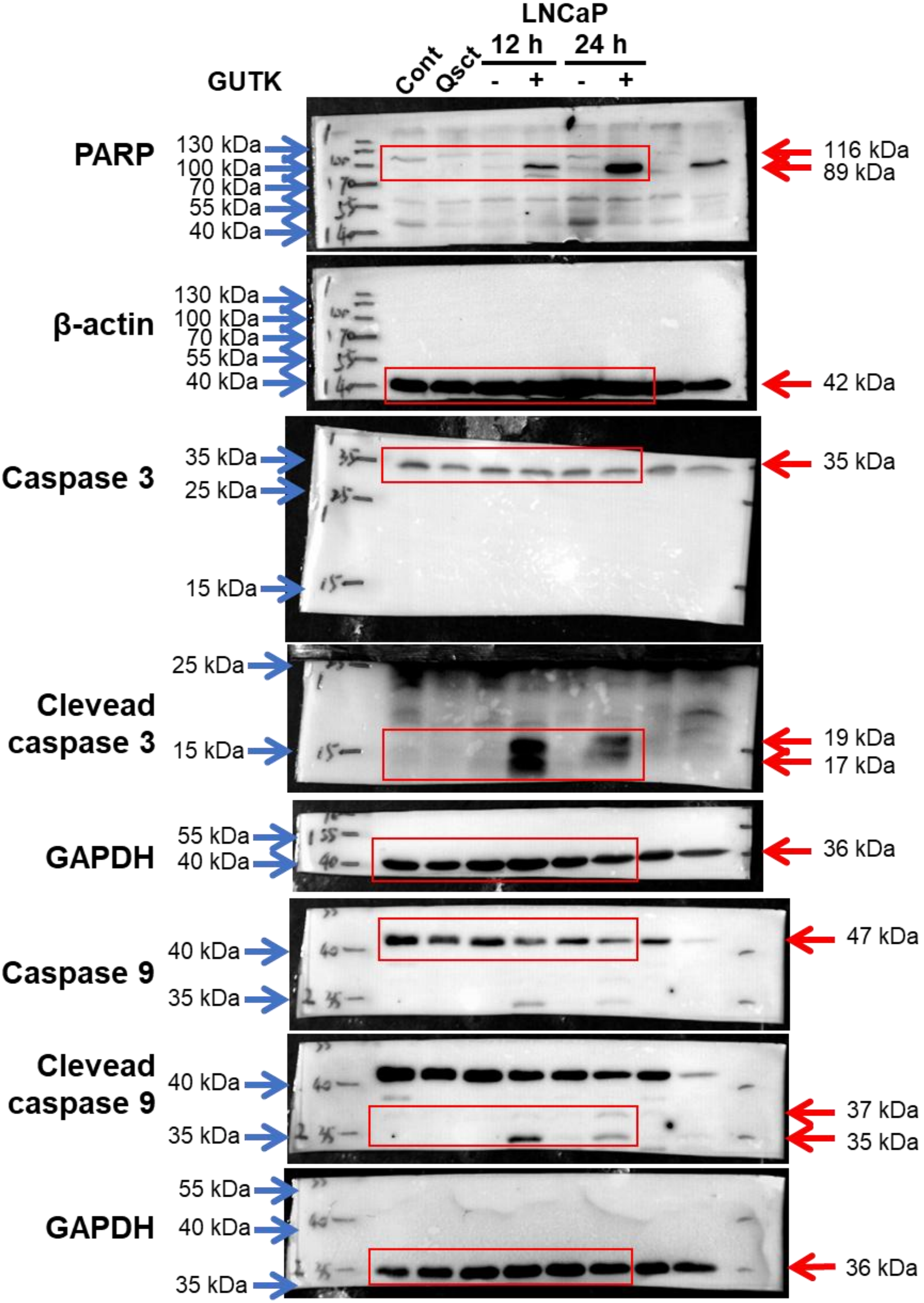

LNCaP
GUTK
Cont
Qsct
12 h
24 h
- + - +
PARP
130 kDa
100 kDa
70 kDa
55 kDa
40 kDa
116 kDa
89 kDa
β-actin
130 kDa
100 kDa
70 kDa
55 kDa
40 kDa
42 kDa
Caspase 3
35 kDa
25 kDa
15 kDa
35 kDa
Clevead caspase 3
25 kDa
15 kDa
19 kDa
17 kDa
GAPDH
55 kDa
40 kDa
36 kDa
Caspase 9
40 kDa
35 kDa
47 kDa
Clevead caspase 9
40 kDa
35 kDa
37 kDa
35 kDa
GAPDH
55 kDa
40 kDa
35 kDa
36 kDa

Figure 1E
N=3

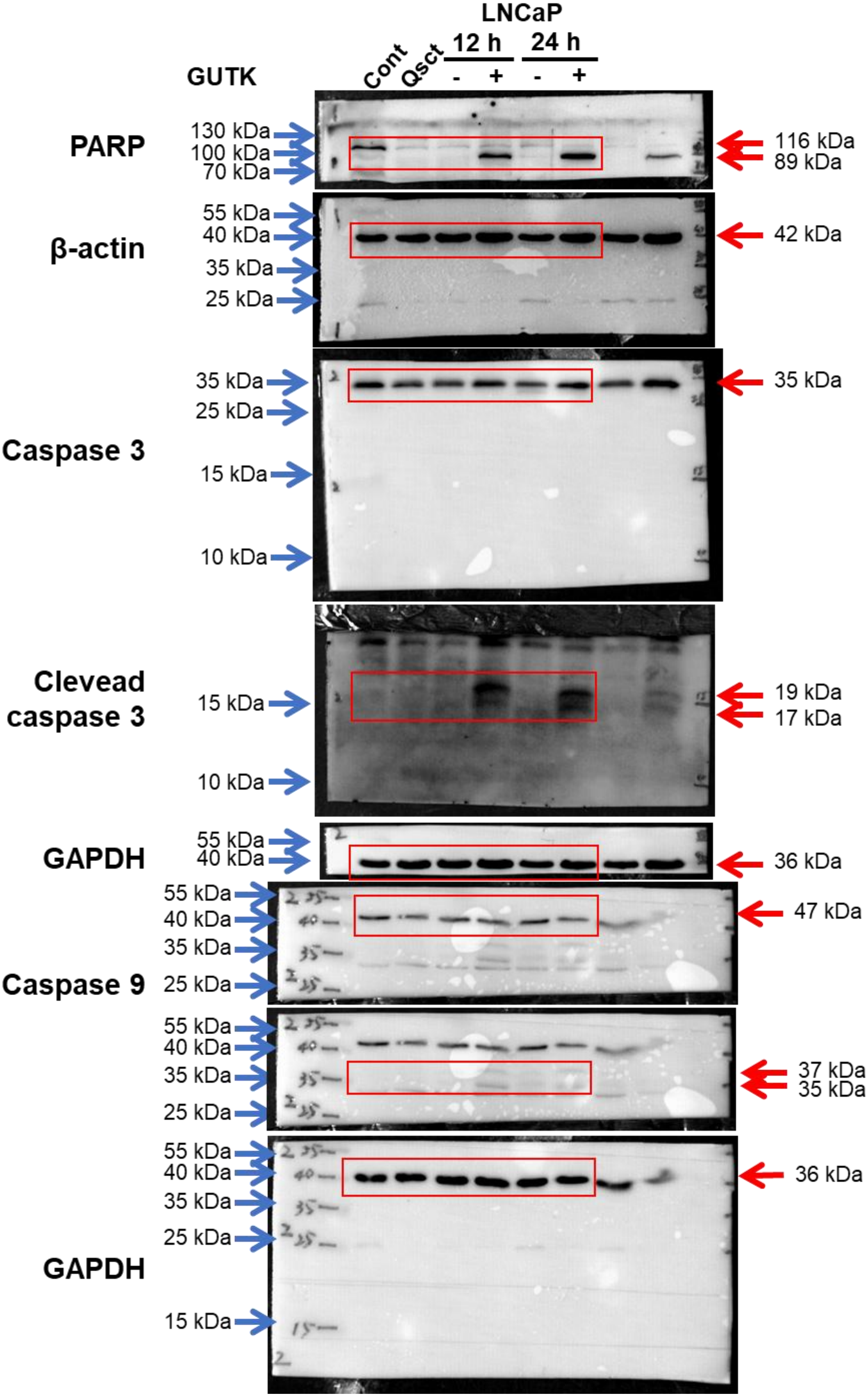

LNCaP
GUTK
Cont
Qsct
12 h
24 h
- + - +
PARP
130 kDa
100 kDa
70 kDa
116 kDa
89 kDa
β-actin
55 kDa
40 kDa
35 kDa
25 kDa
42 kDa
Caspase 3
35 kDa
25 kDa
15 kDa
10 kDa
35 kDa
Clevead caspase 3
15 kDa
10 kDa
19 kDa
17 kDa
GAPDH
55 kDa
40 kDa
36 kDa
Caspase 9
55 kDa
40 kDa
35 kDa
25 kDa
47 kDa
55 kDa
40 kDa
35 kDa
25 kDa
37 kDa
35 kDa
GAPDH
55 kDa
40 kDa
35 kDa
25 kDa
15 kDa
36 kDa

Figure 1F
N=1

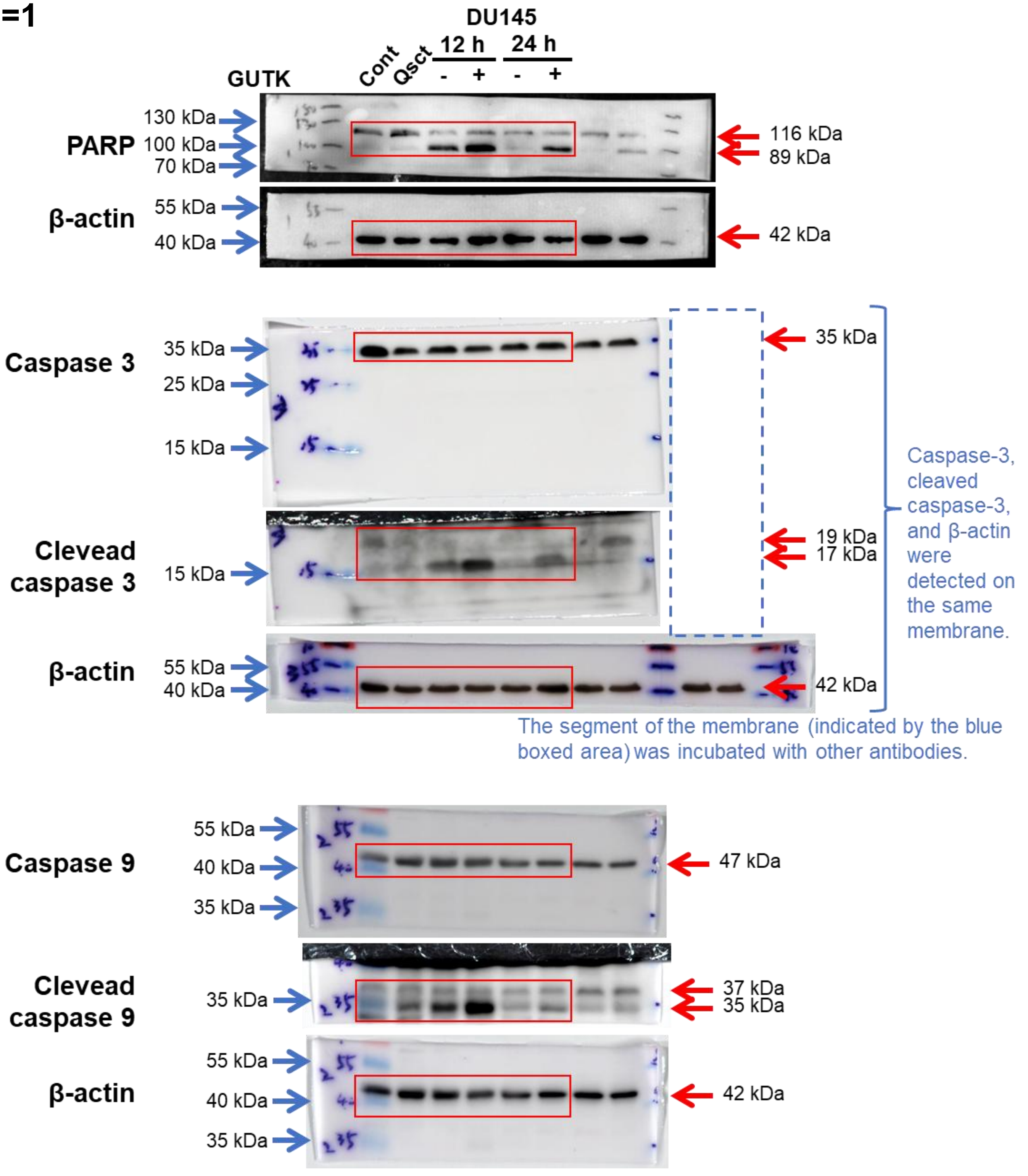

DU145
12 h
24 h
GUTK
Cont
Qsct
-
+
-
+
PARP
130 kDa
100 kDa
70 kDa
116 kDa
89 kDa
β-actin
55 kDa
40 kDa
42 kDa
Caspase 3
35 kDa
25 kDa
15 kDa
35 kDa
Clevead caspase 3
15 kDa
19 kDa
17 kDa
β-actin
55 kDa
40 kDa
42 kDa
Caspase-3, cleaved caspase-3, and β-actin were detected on the same membrane.
The segment of the membrane (indicated by the blue boxed area) was incubated with other antibodies.
Caspase 9
55 kDa
40 kDa
35 kDa
47 kDa
Clevead caspase 9
35 kDa
37 kDa
35 kDa
β-actin
55 kDa
40 kDa
35 kDa
42 kDa

Figure 1F
N=2

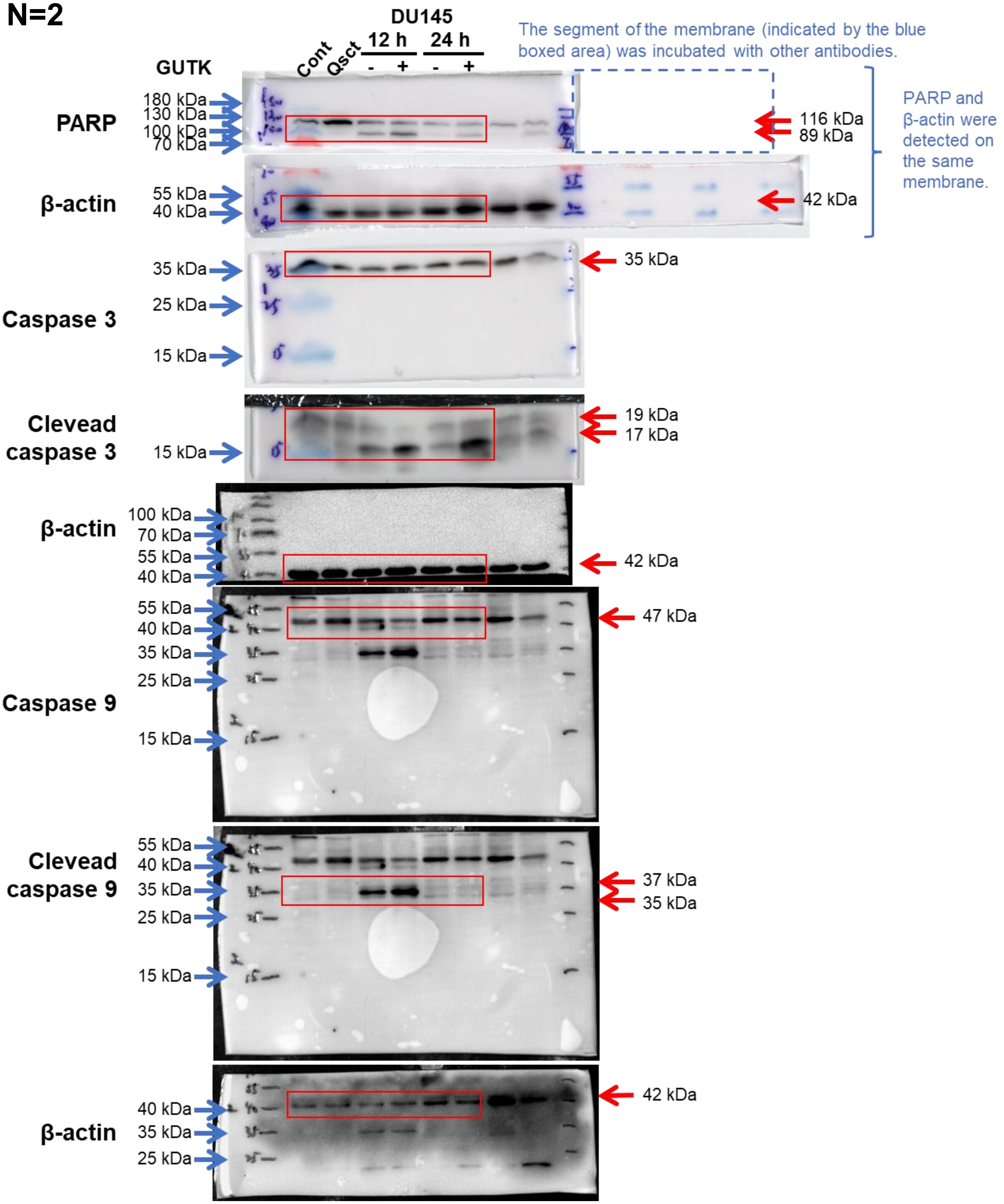
DU145
12 h
24 h
Cont
Qsct
- + - +
GUTK
The segment of the membrane (indicated by the blue boxed area) was incubated with other antibodies.
PARP and β-actin were detected on the same membrane.
PARP
180 kDa
130 kDa
100 kDa
70 kDa
116 kDa
89 kDa
β-actin
55 kDa
40 kDa
42 kDa
Caspase 3
35 kDa
25 kDa
15 kDa
35 kDa
Clevead caspase 3
15 kDa
19 kDa
17 kDa
β-actin
100 kDa
70 kDa
55 kDa
40 kDa
42 kDa
Caspase 9
55 kDa
40 kDa
35 kDa
25 kDa
15 kDa
47 kDa
Clevead caspase 9
55 kDa
40 kDa
35 kDa
25 kDa
15 kDa
37 kDa
35 kDa
β-actin
40 kDa
35 kDa
25 kDa
42 kDa

Figure 1F
N=3

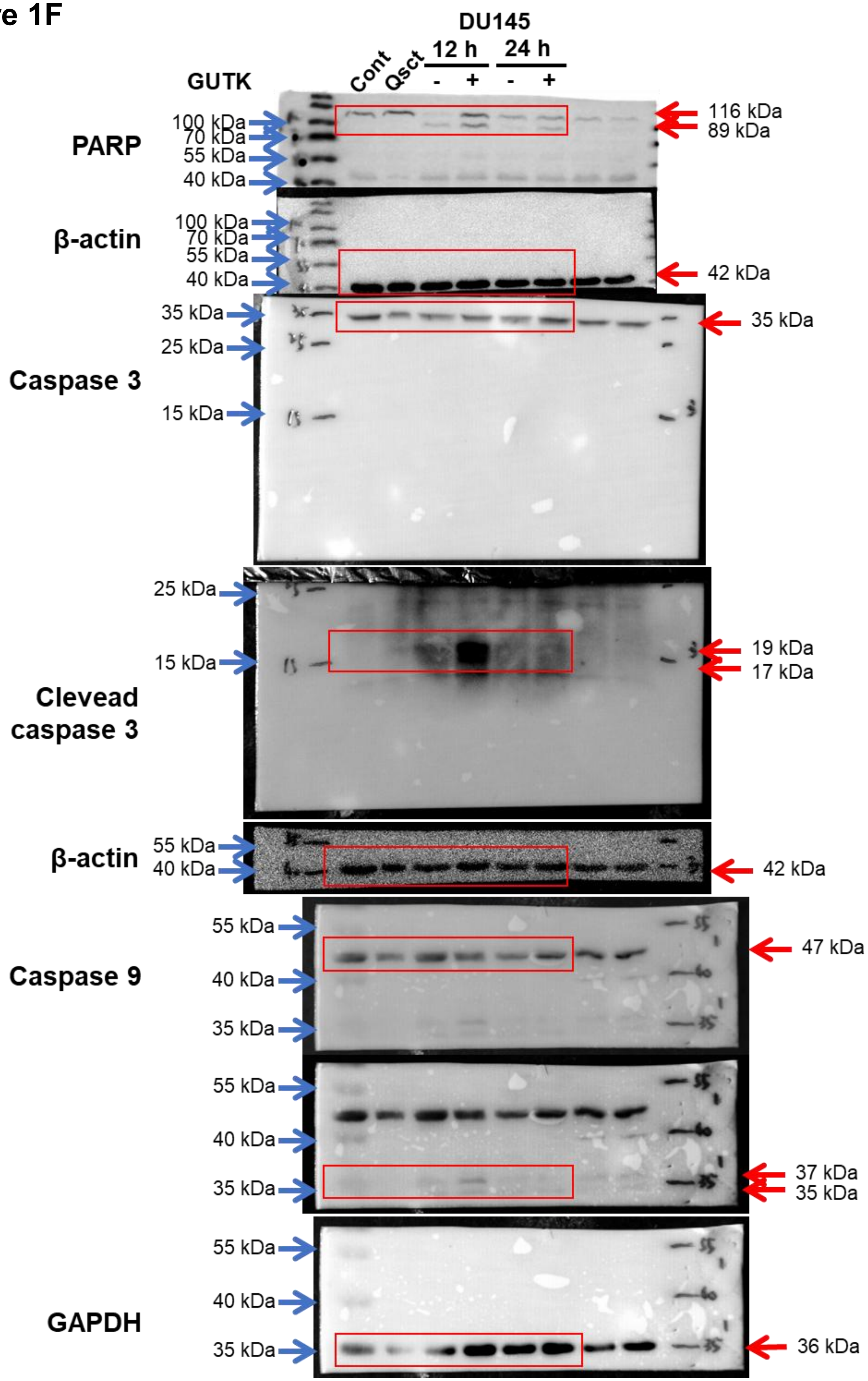

DU145
12 h
24 h
Cont
Qsct
-
+
-
+
GUTK
PARP
100 kDa
70 kDa
55 kDa
40 kDa
116 kDa
89 kDa
β-actin
100 kDa
70 kDa
55 kDa
40 kDa
42 kDa
Caspase 3
35 kDa
25 kDa
15 kDa
35 kDa
Clevead caspase 3
25 kDa
15 kDa
19 kDa
17 kDa
β-actin
55 kDa
40 kDa
42 kDa
Caspase 9
55 kDa
40 kDa
35 kDa
47 kDa
55 kDa
40 kDa
35 kDa
37 kDa
35 kDa
GAPDH
55 kDa
40 kDa
35 kDa
36 kDa

Figure 2A
N=1

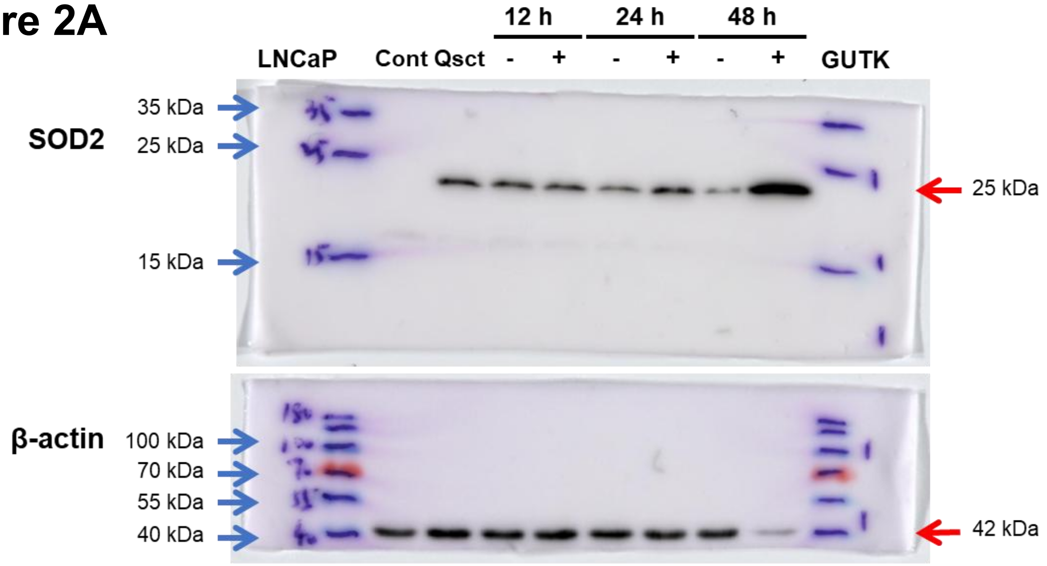

Figure 2A
N=2

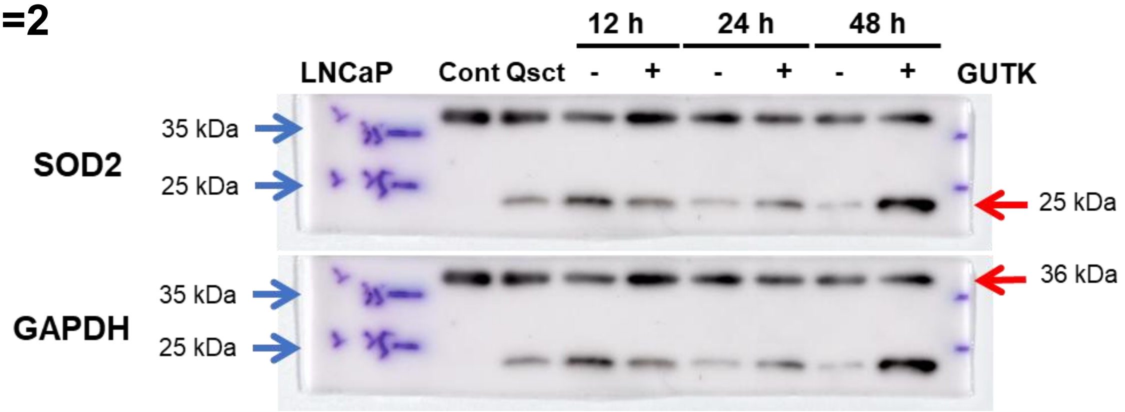

Figure 2A
N=3

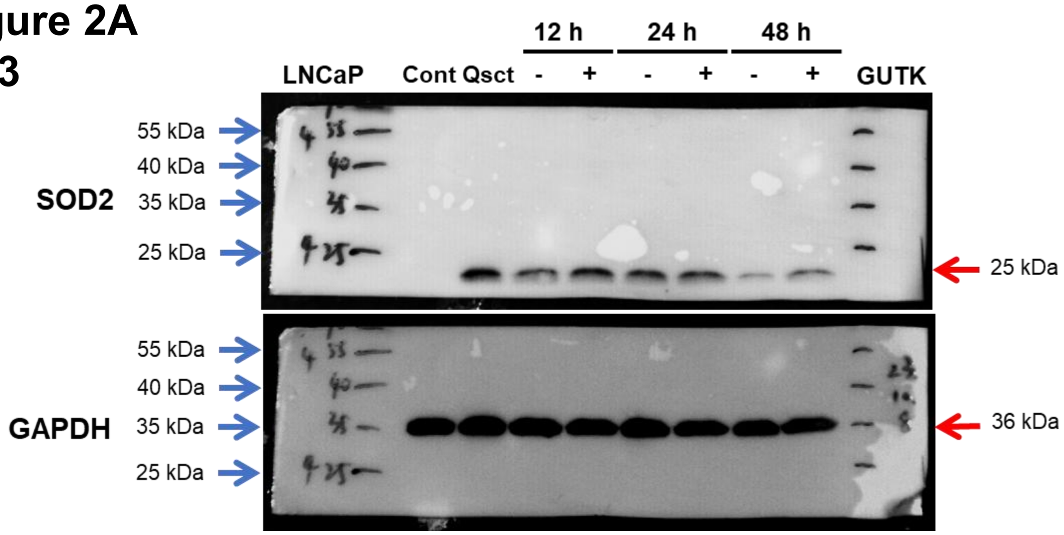

Figure 2B
N=1

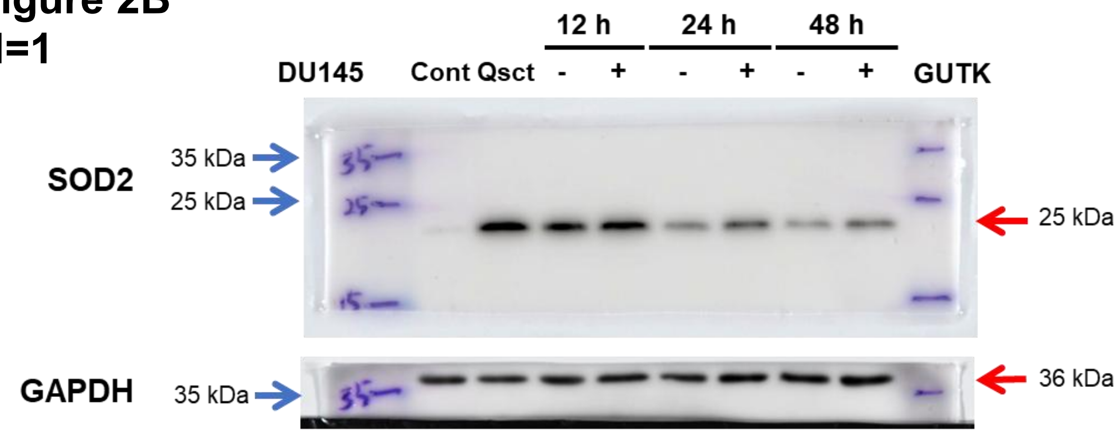

Figure 2B
N=2

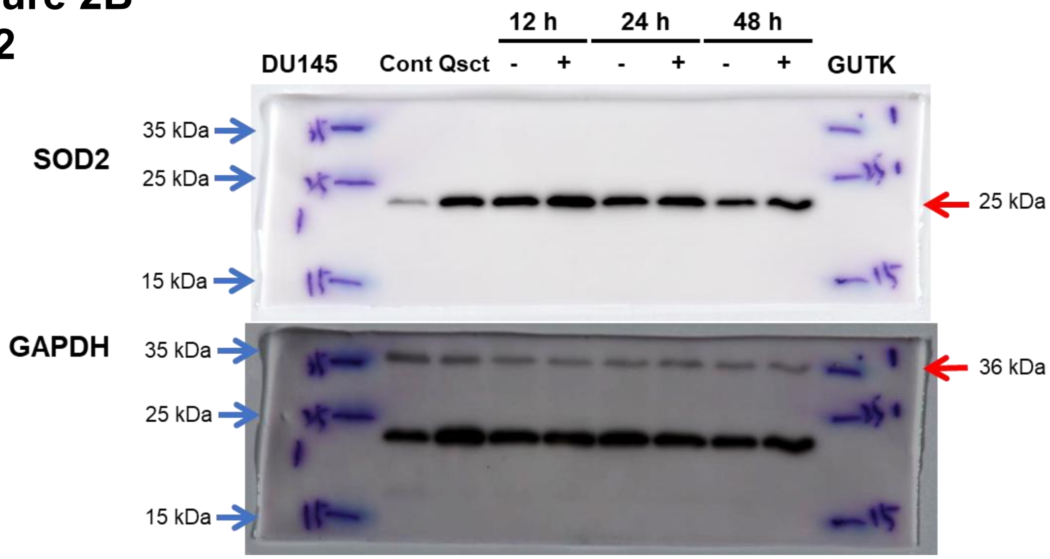

Figure 2B
N=3

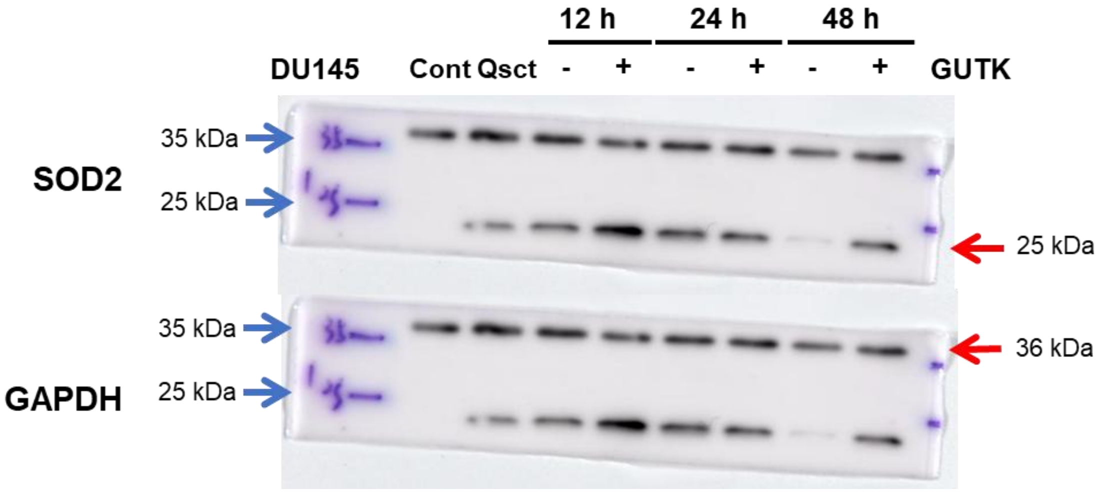

Figure 2C
N=1

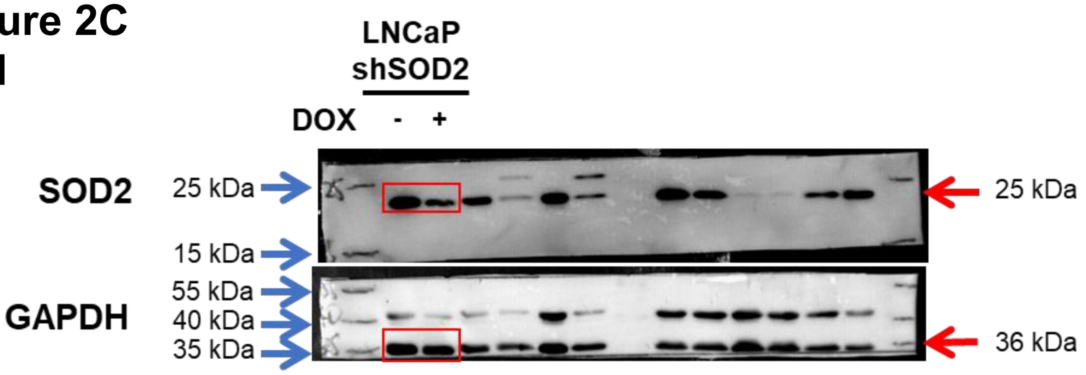

Figure 2C
N=2

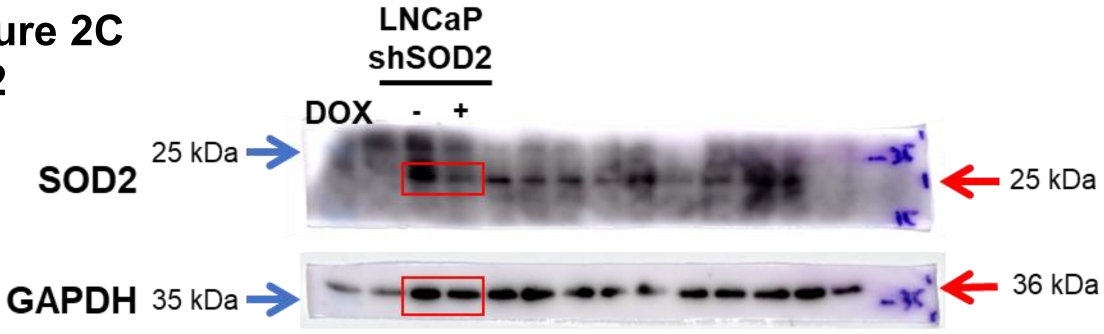

Figure 2C
N=3

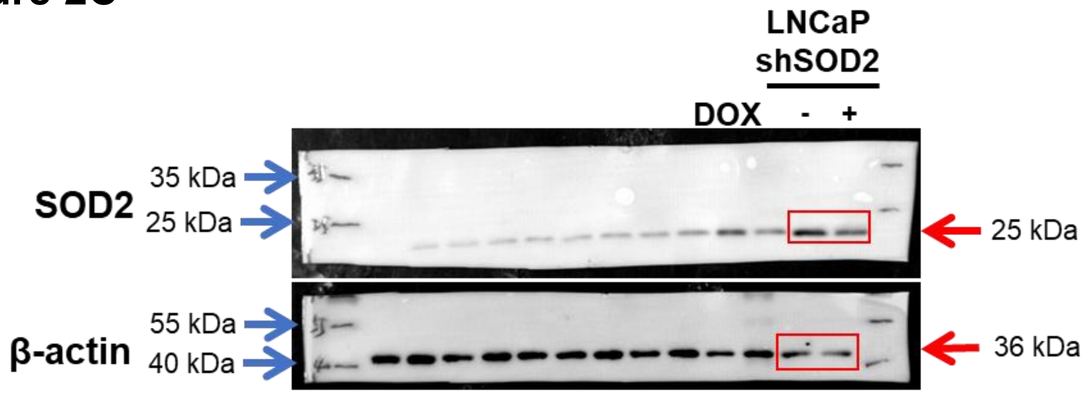

Figure 2D
N=1

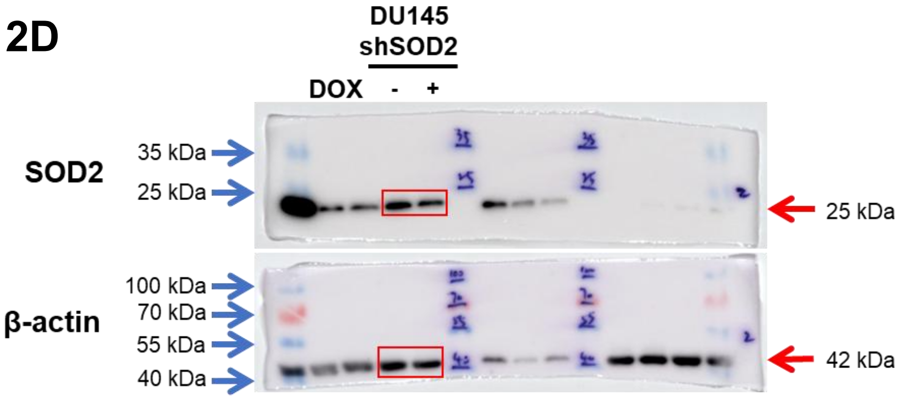

Figure 2D
N=2

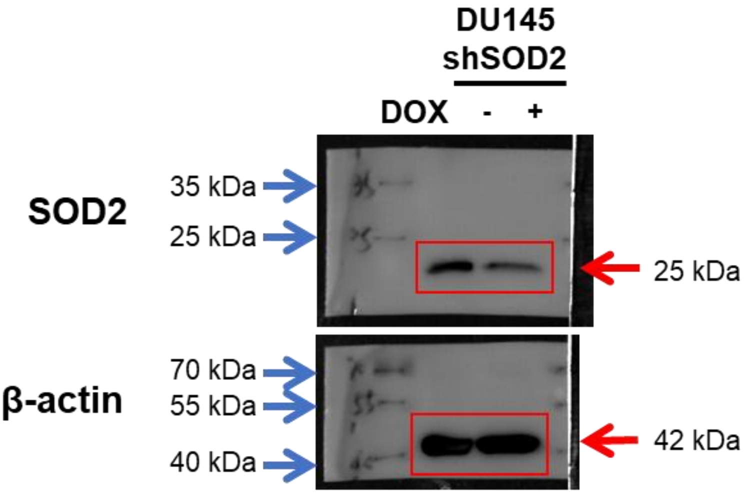

Figure 2D
N=3

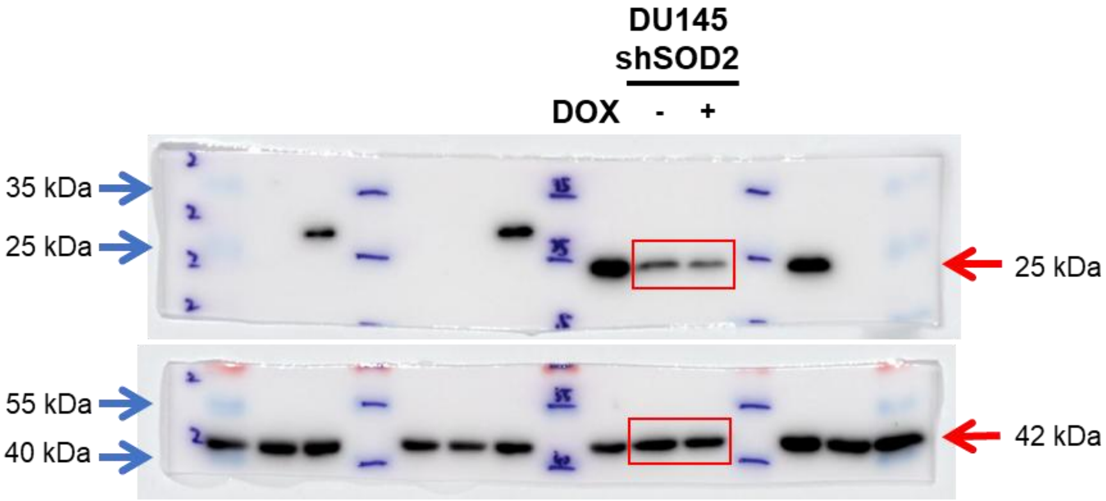

Figure 3A
N=1

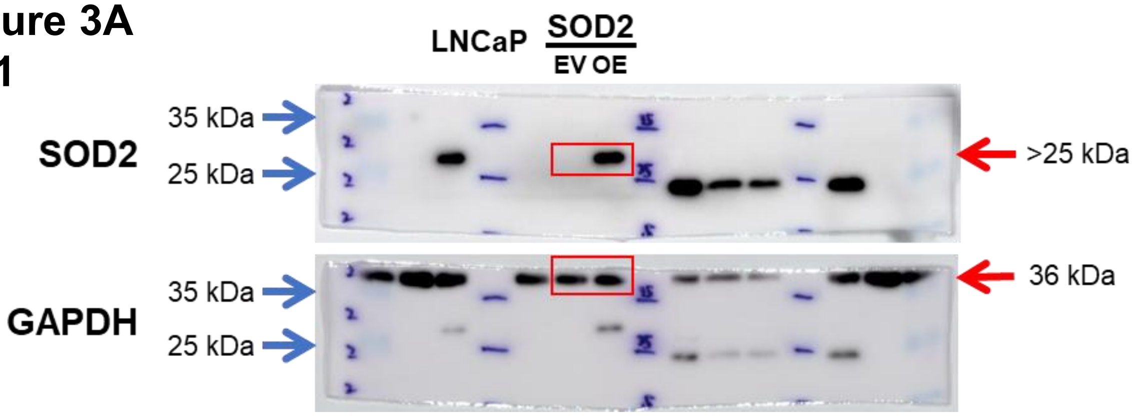

Figure 3A
N=2

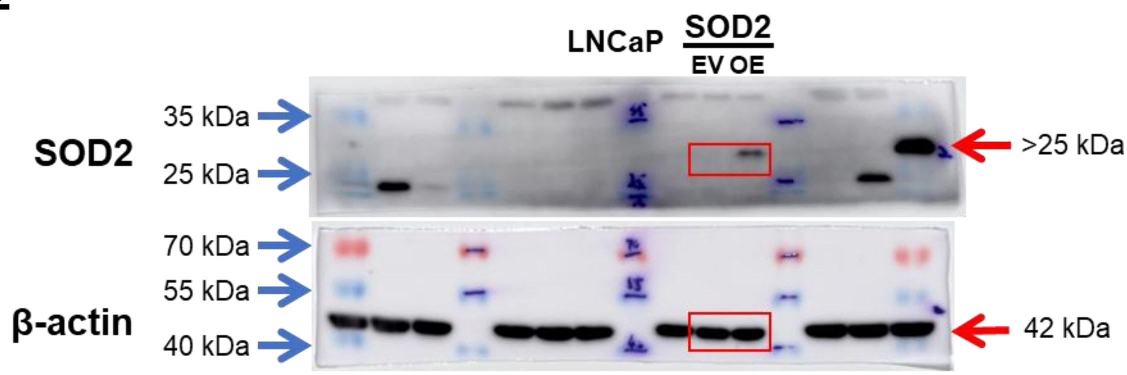

Figure 3A
N=3

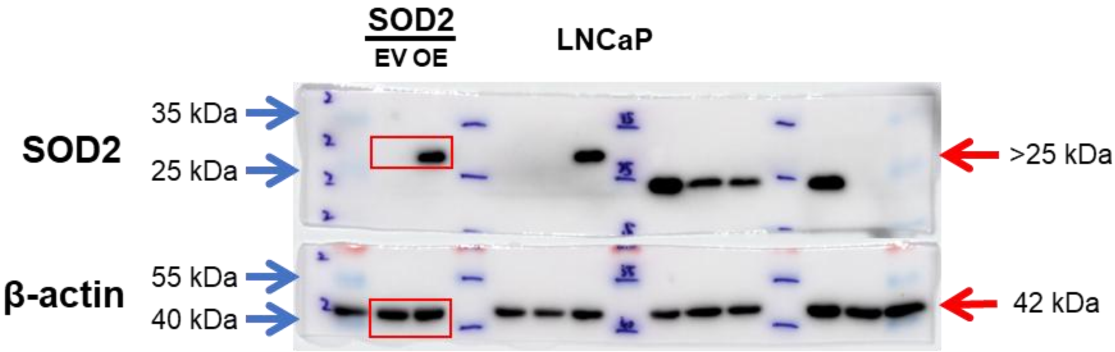

Figure 3B
N=1

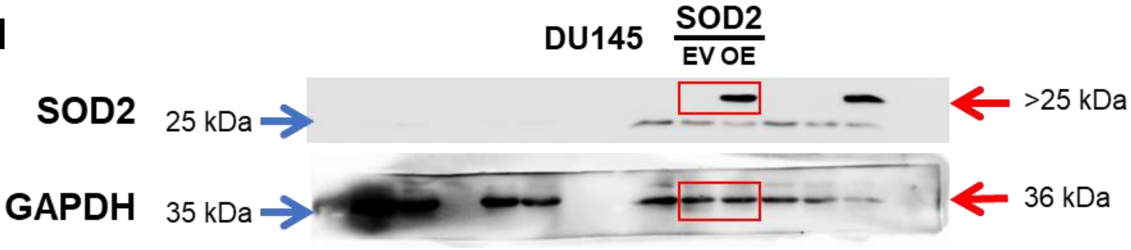

Figure 3B
N=2

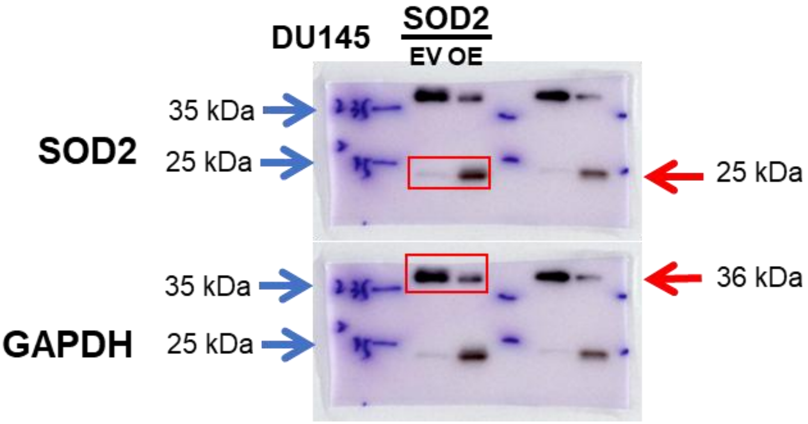

Figure 3B
N=3

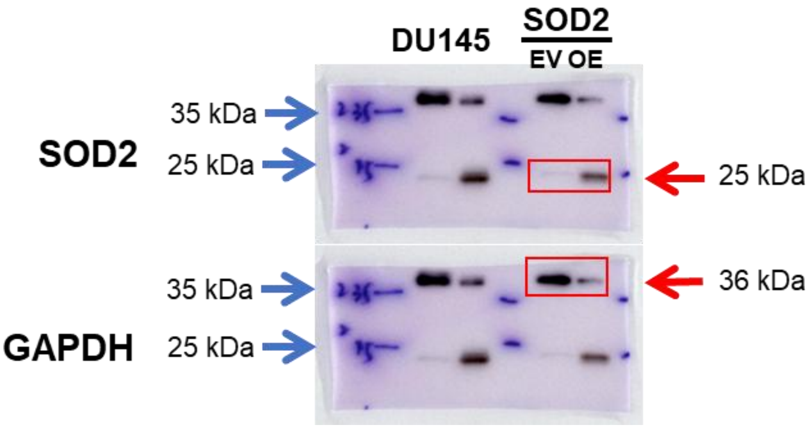

Figure 4C
N=1

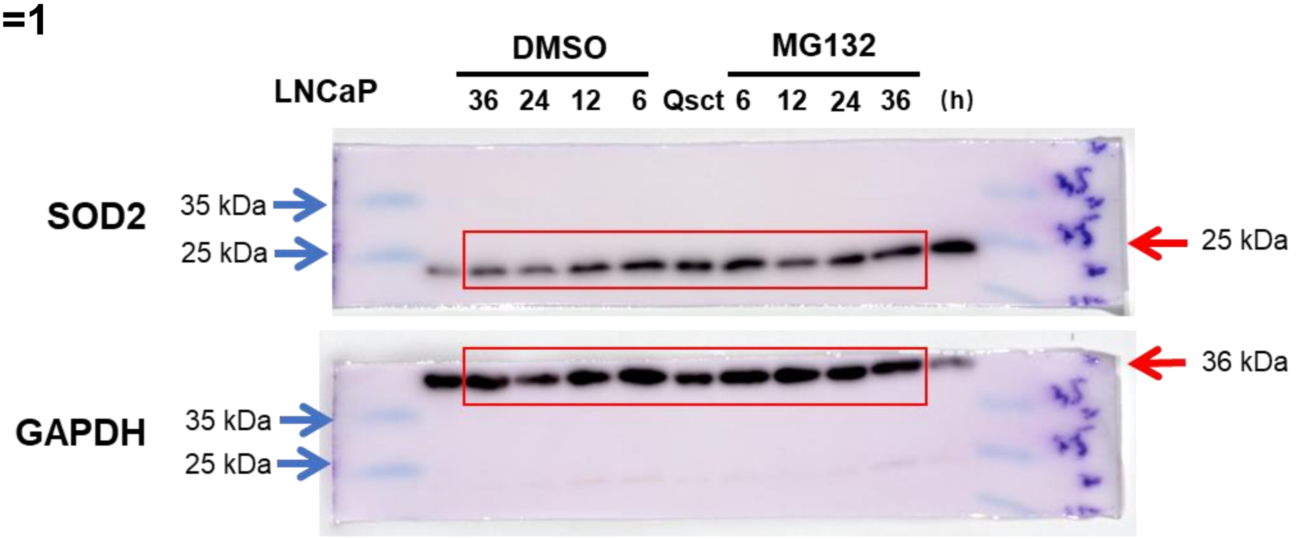

Figure 4C
N=2

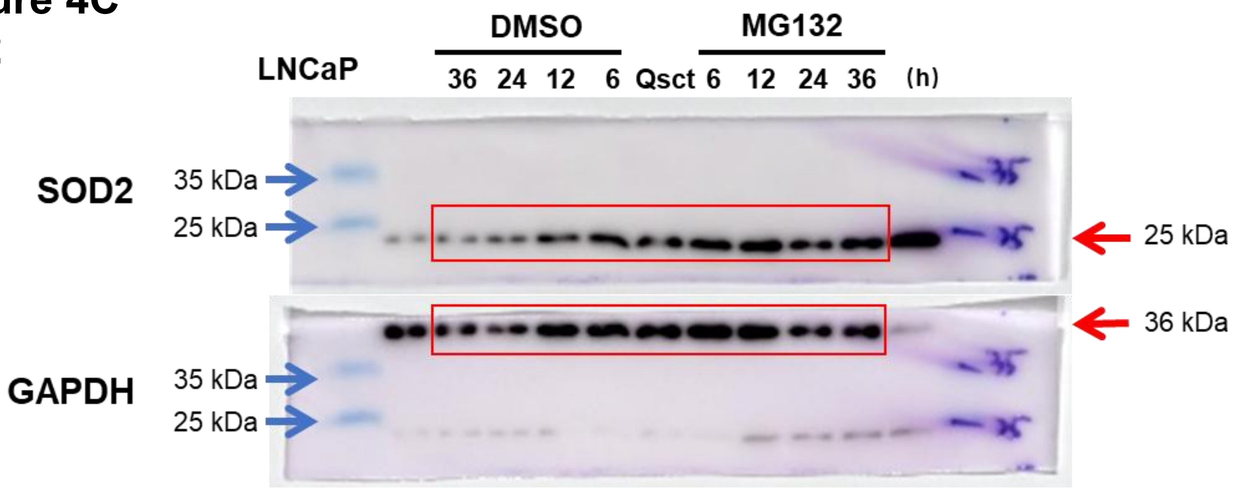

Figure 4C
N=2

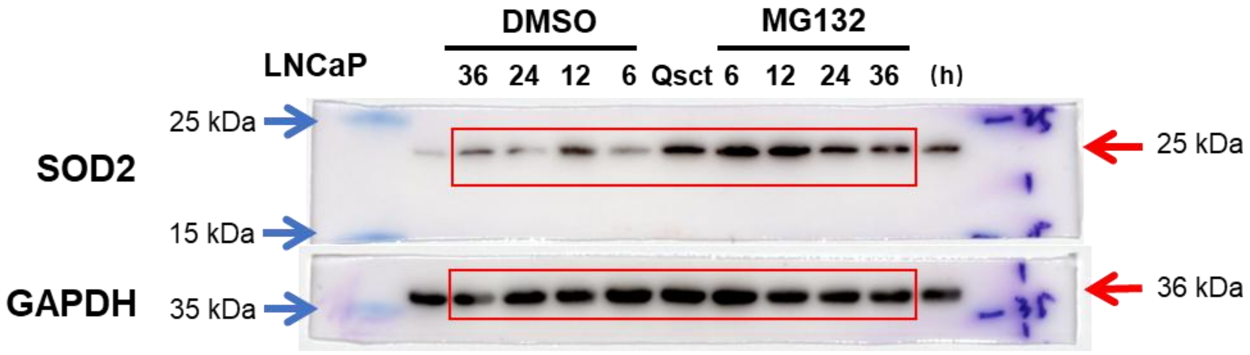

Figure 4D
N=1

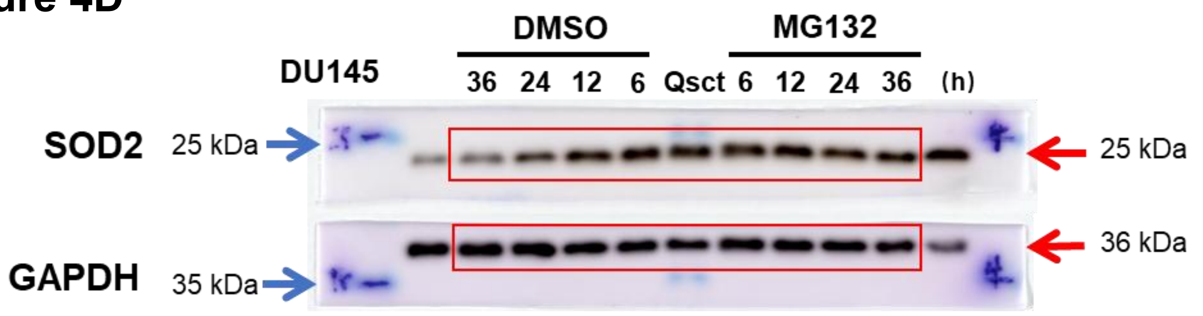

Figure 4D
N=2

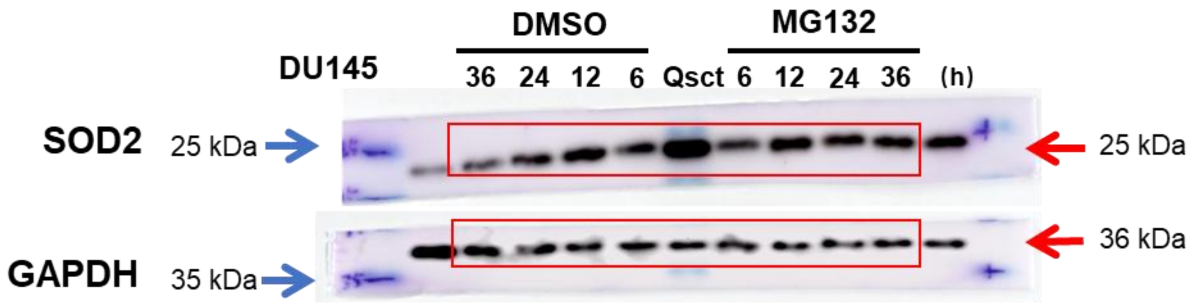

Figure 4E
N=1

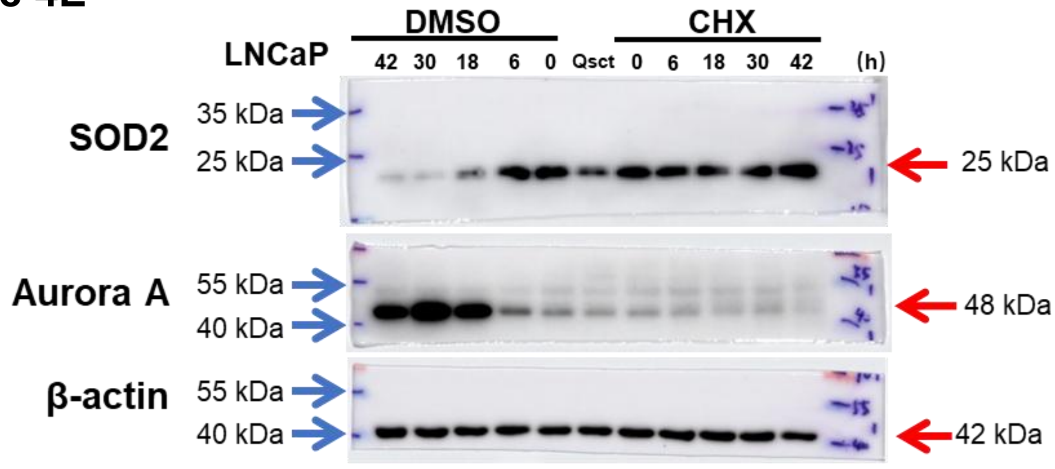

Figure 4E
N=2

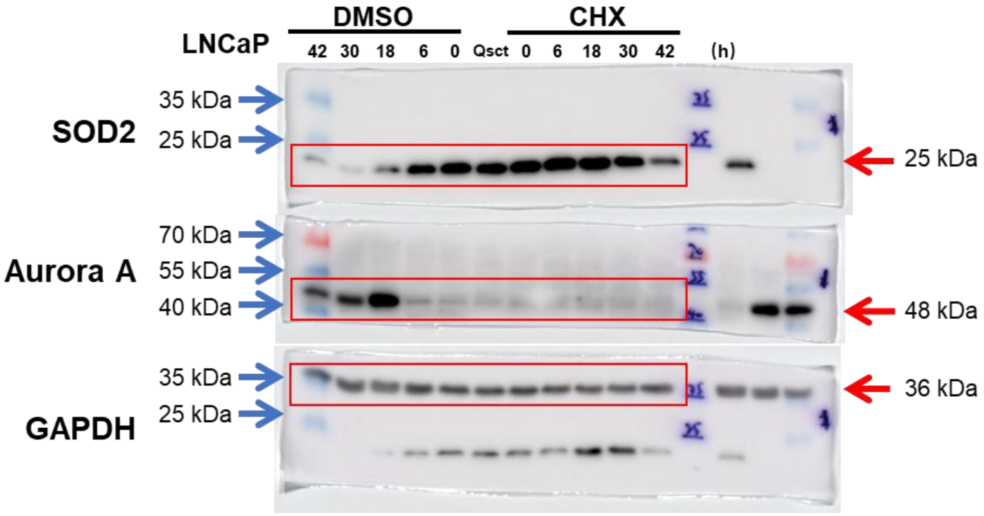

Figure 4E
N=3

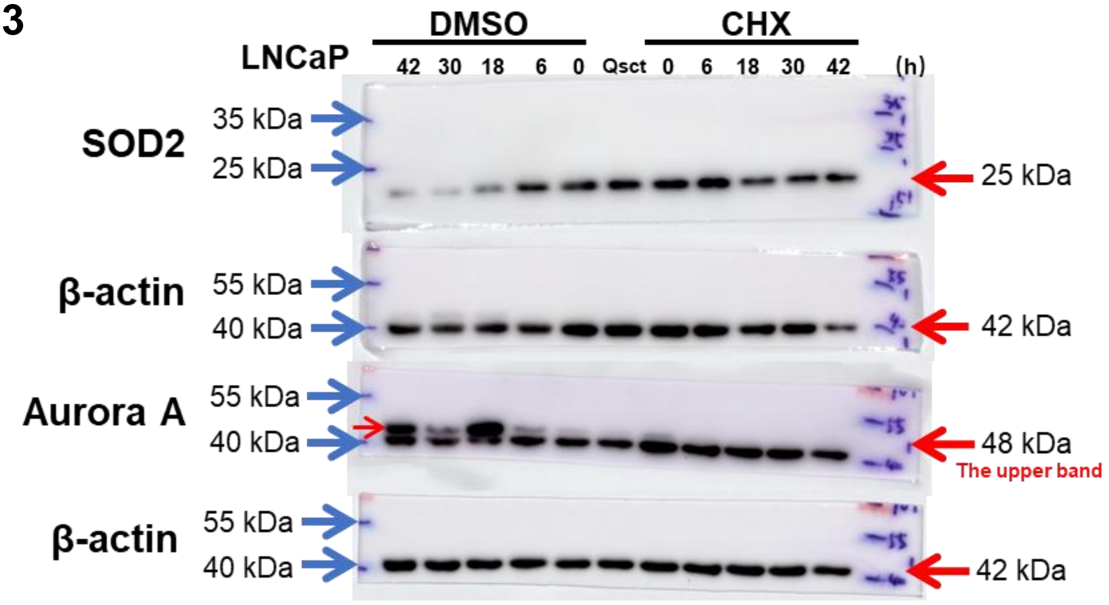

Figure 4F
N=1

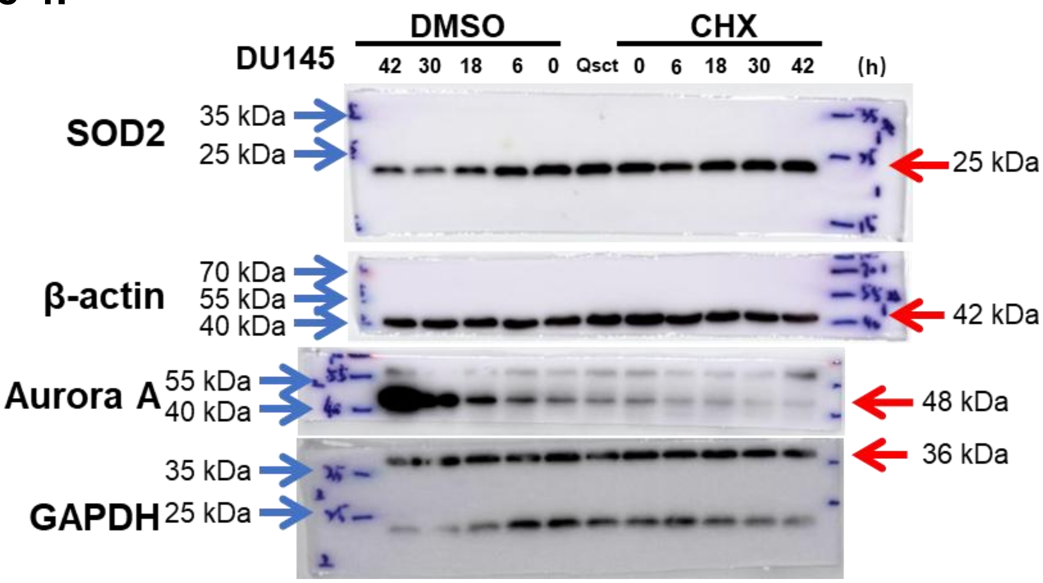

Figure 4F
N=2

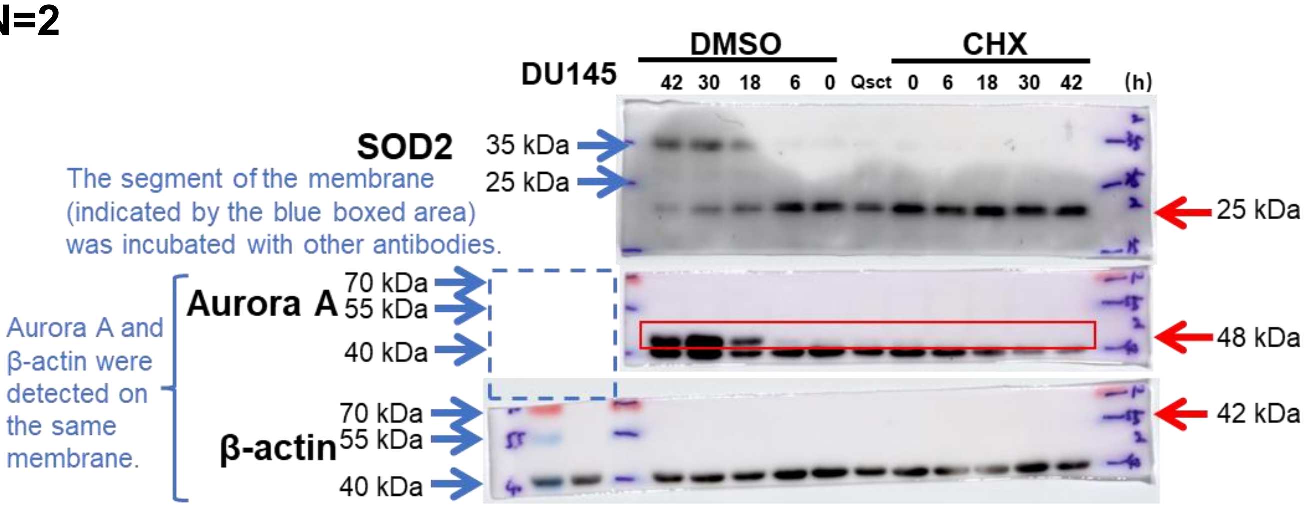

Figure 4F
N=3

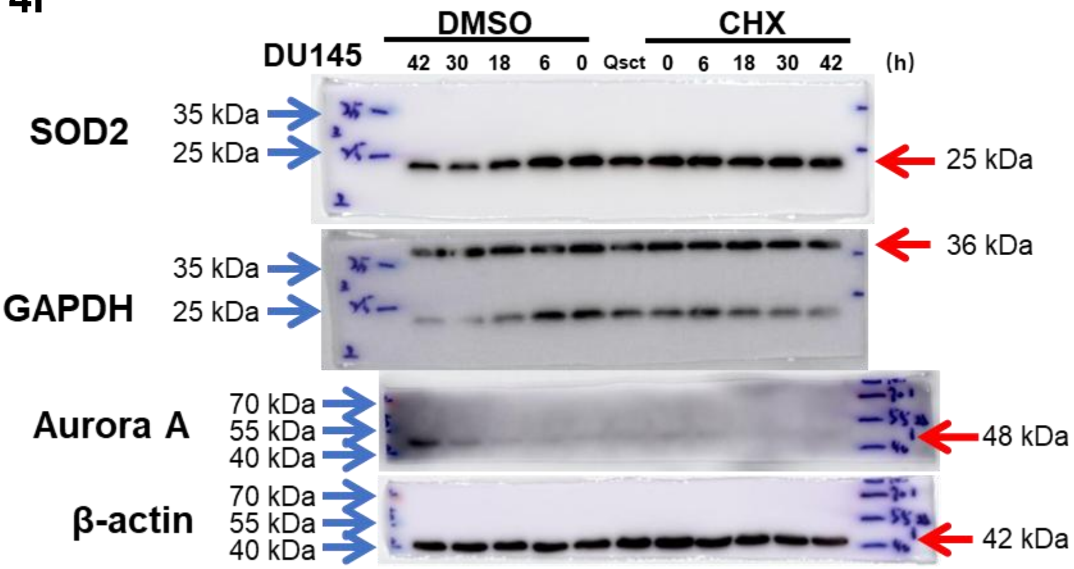

Figure 4G
N=1

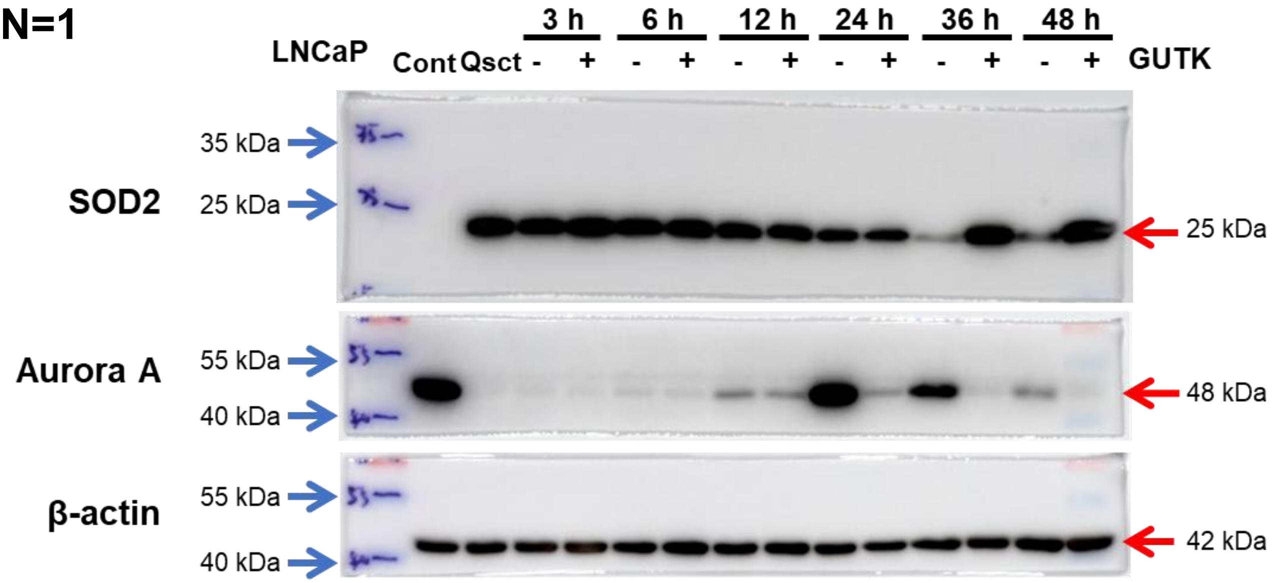

Figure 4G
N=2

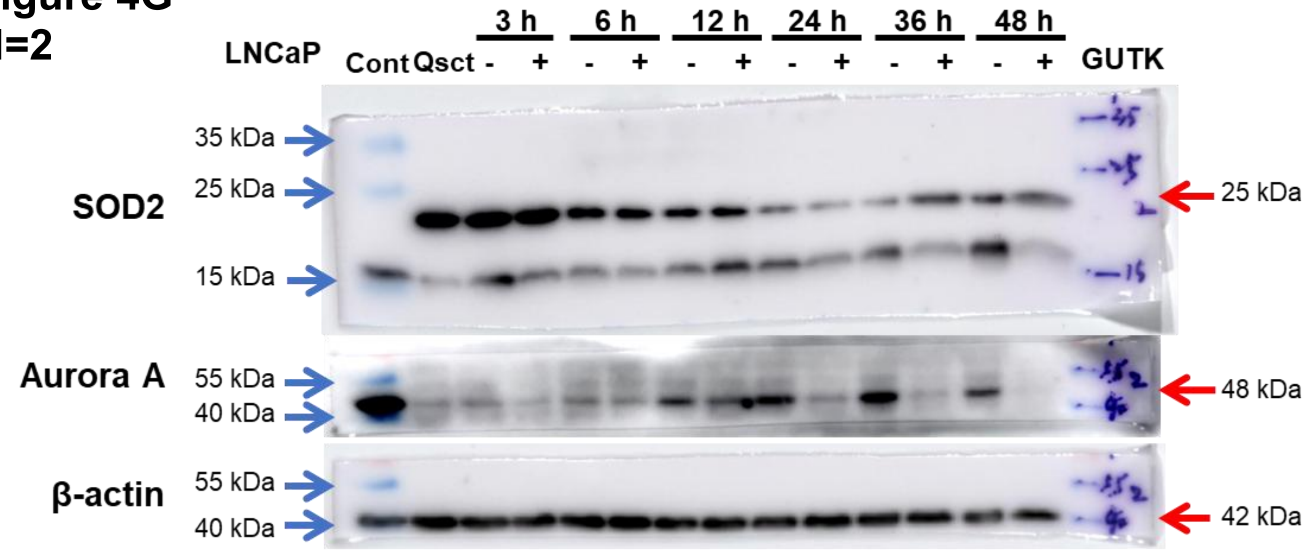

Figure 4G
N=3

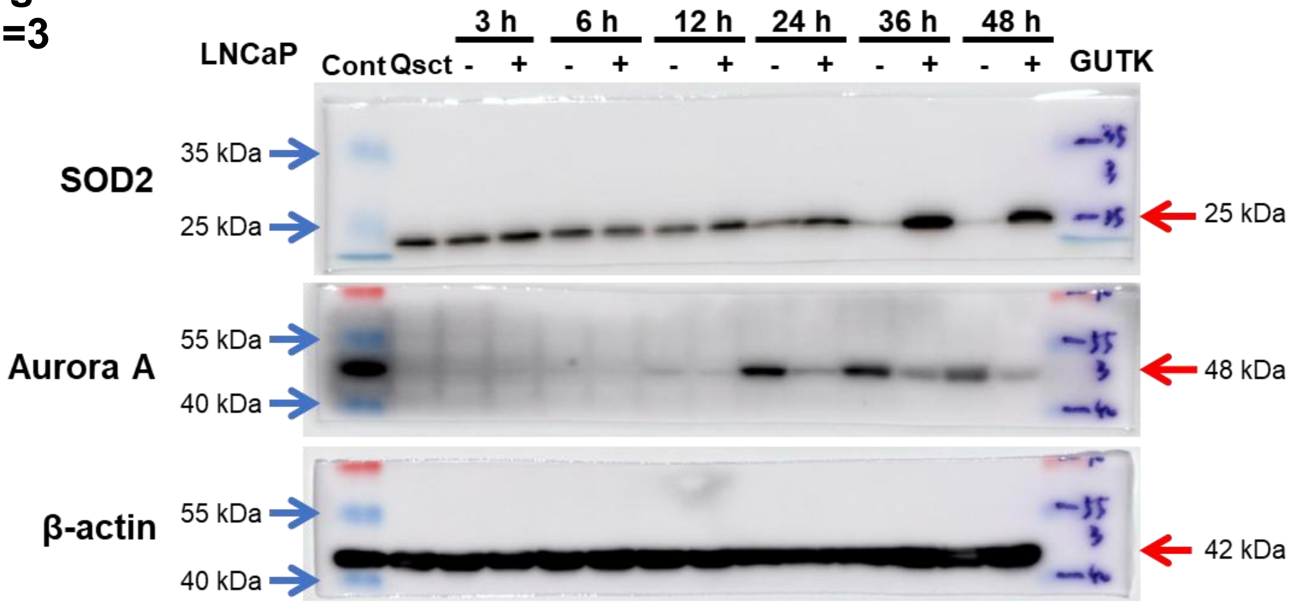

Figure 4H
N=1

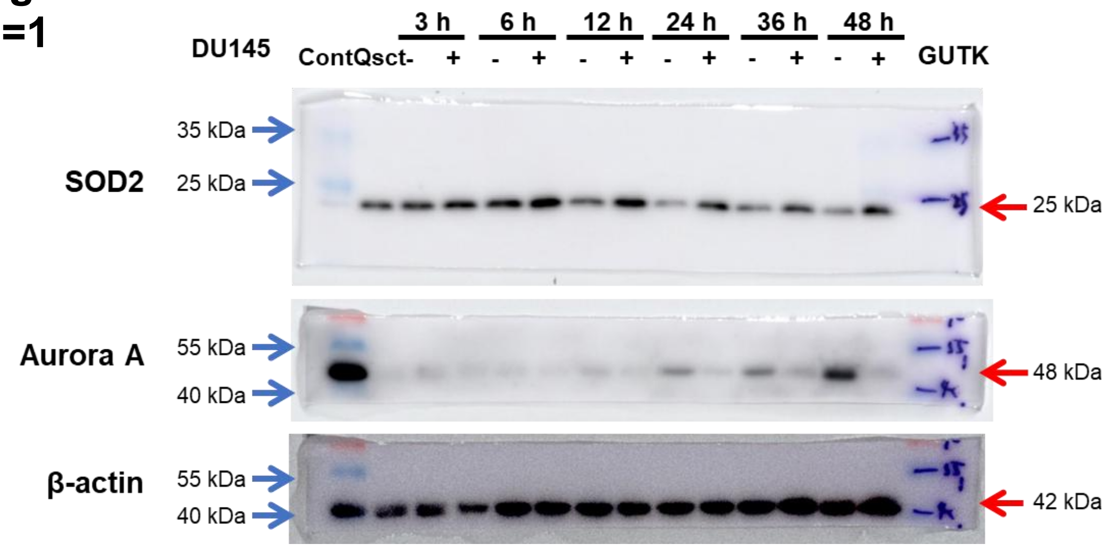

Figure 4H
N=2

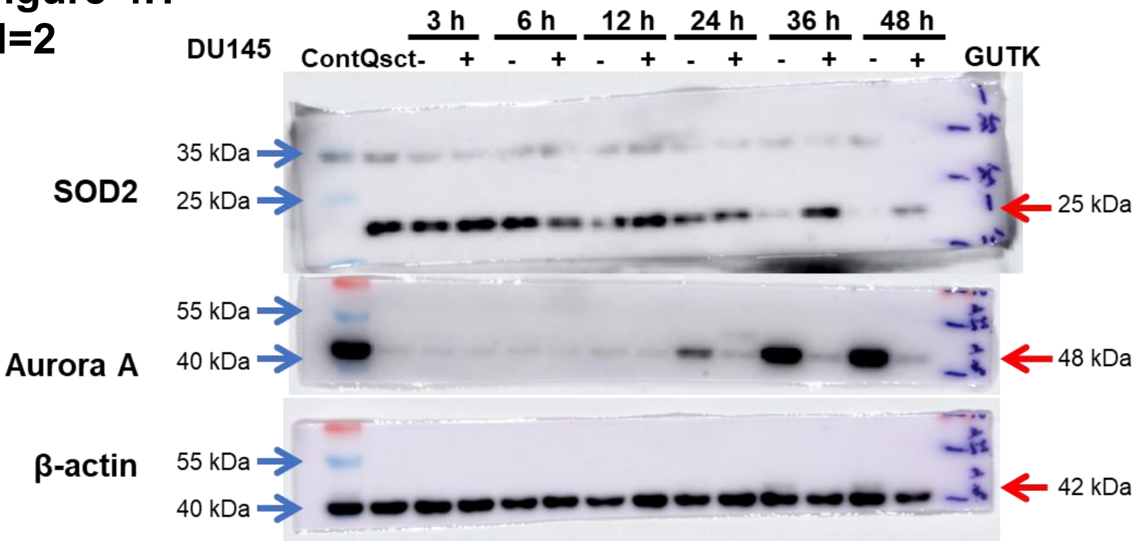

Figure 4H
N=3

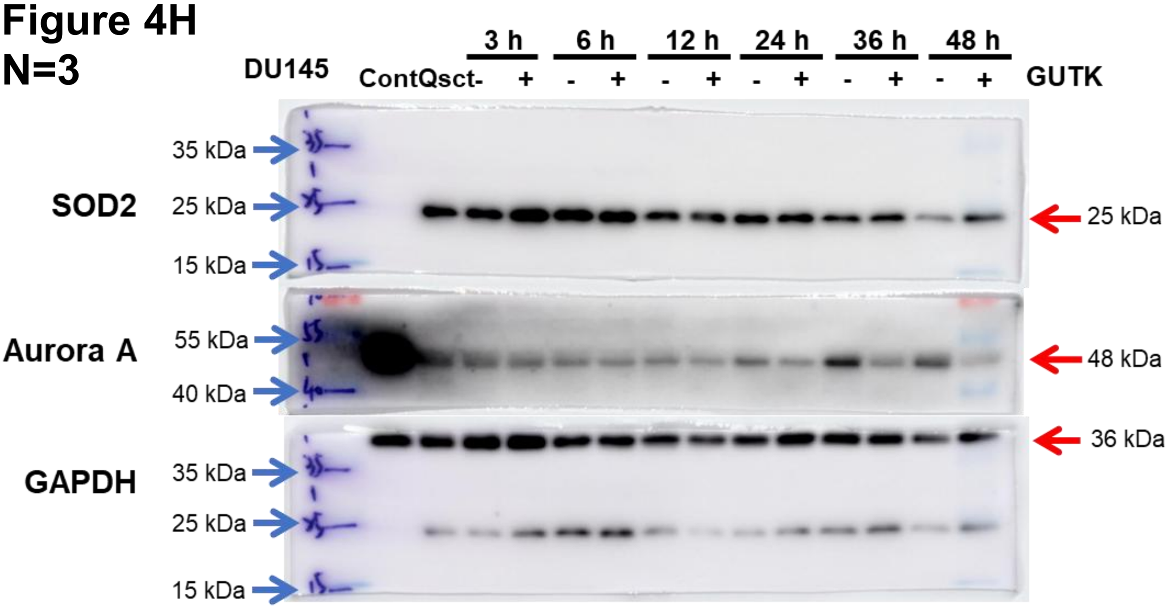

Figure 5C
N=1

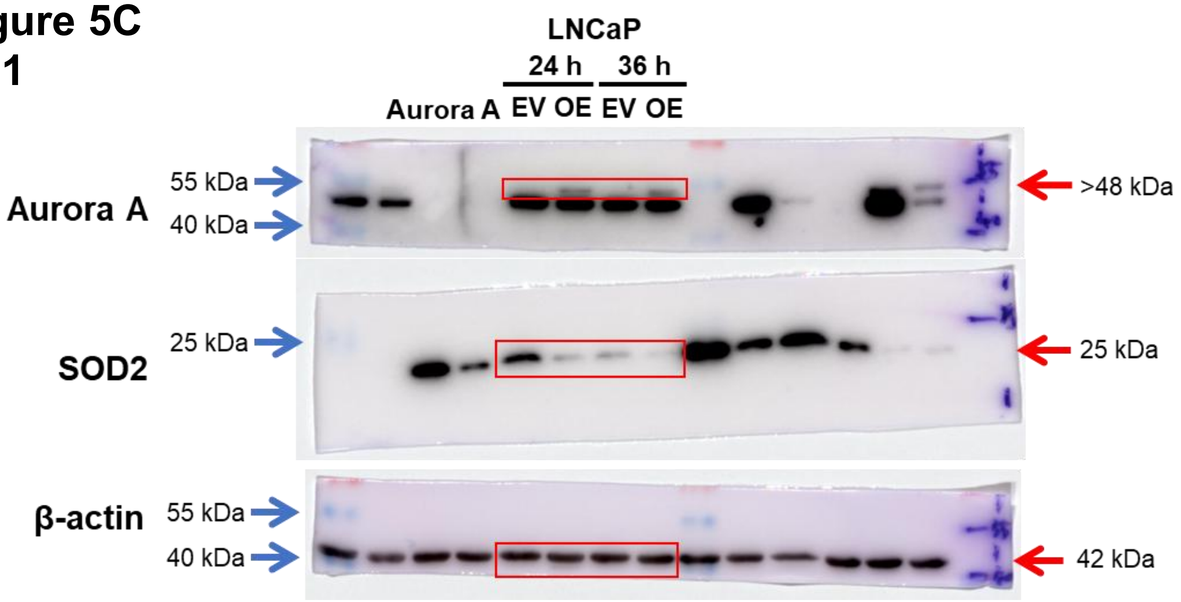

Figure 5C
N=2

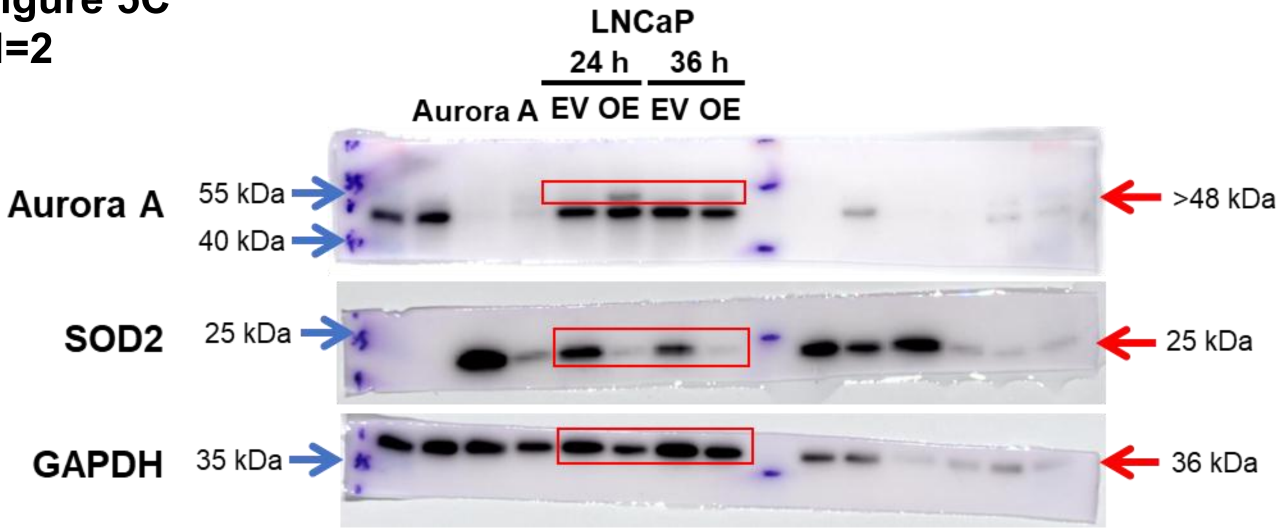

Figure 5C
N=3

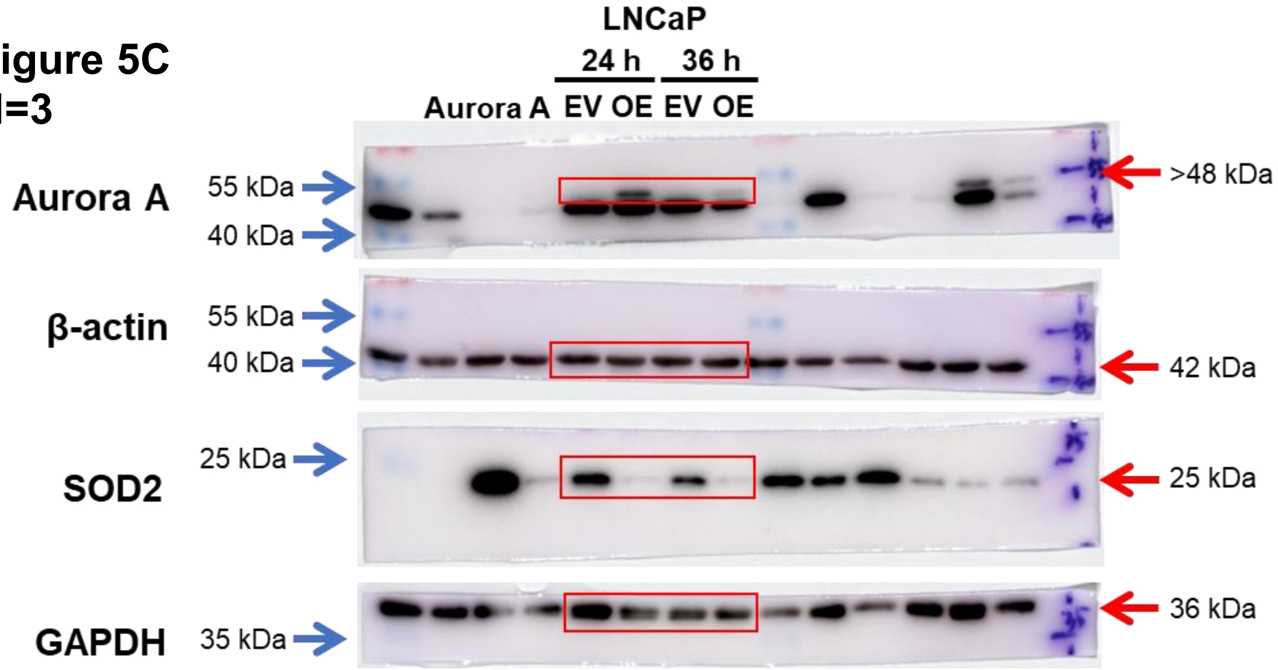

Figure 5D
N=1

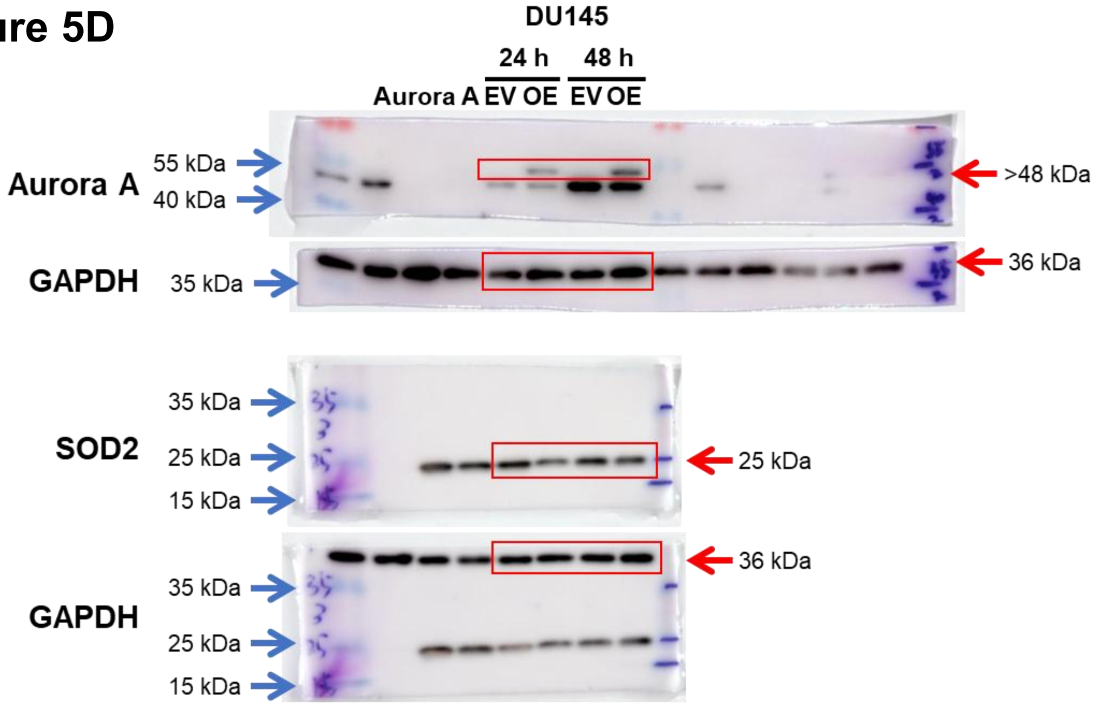

Figure 5D
N=2

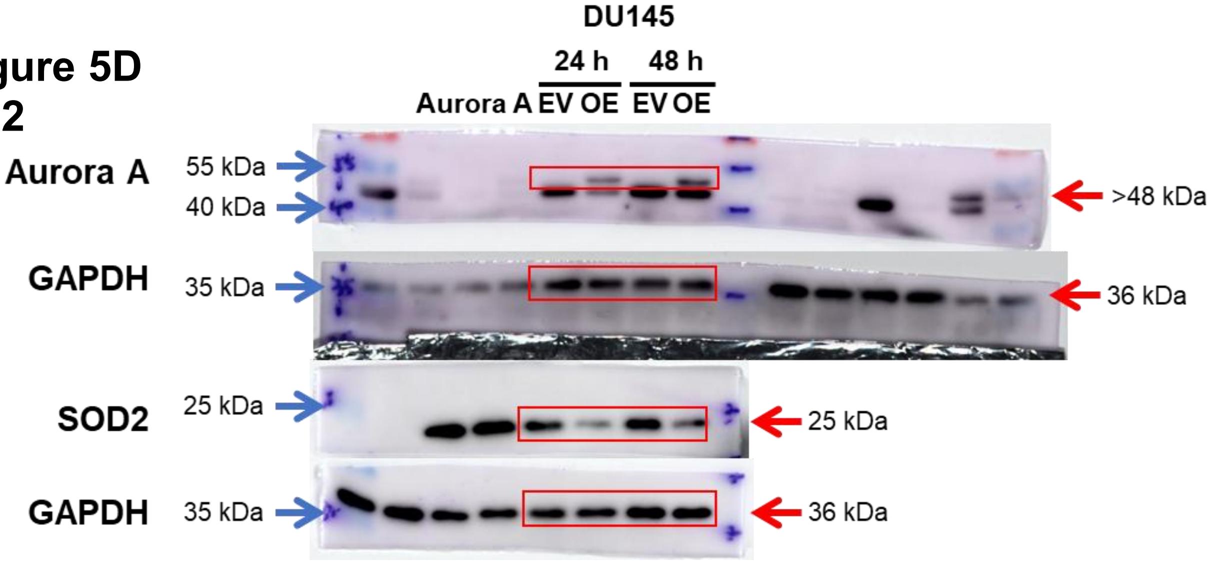

Figure 5D
N=3

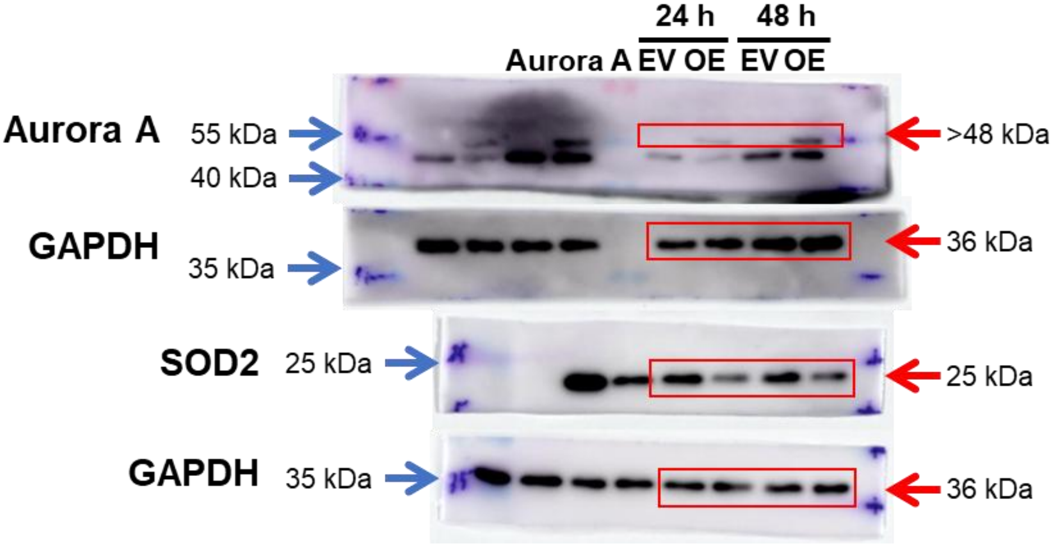

Figure S1C
N=1

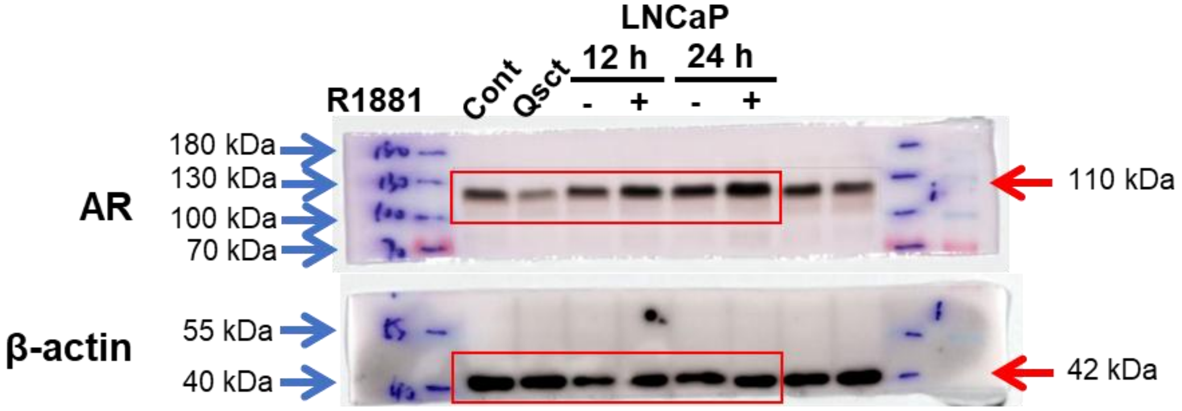

Figure S1C
N=2

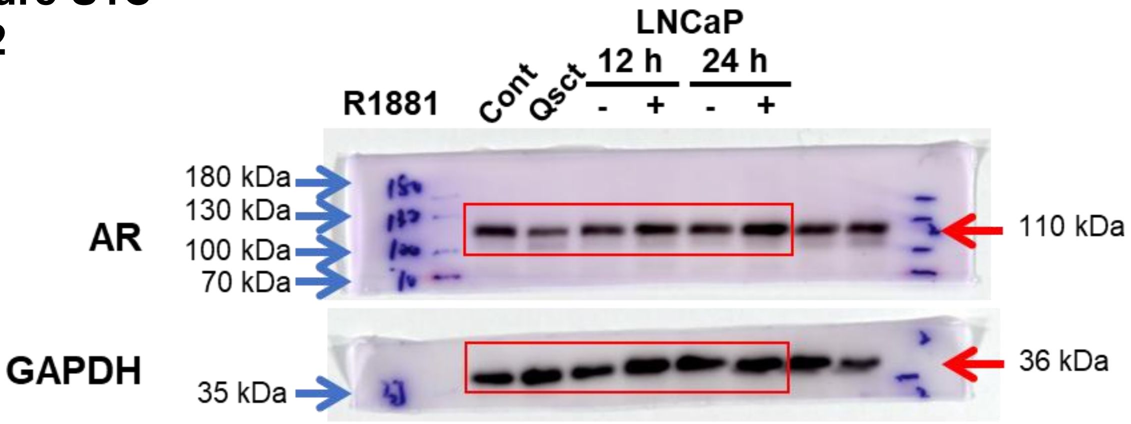

Figure S1D
N=1

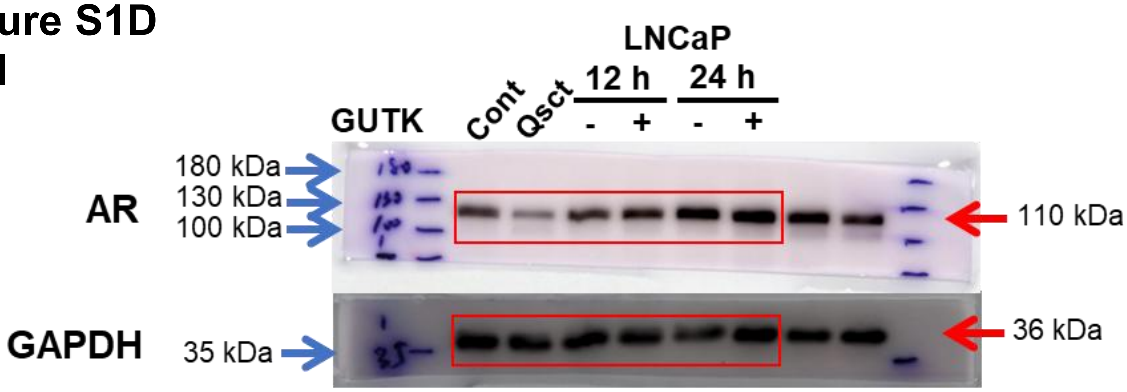

Figure S1D
N=2

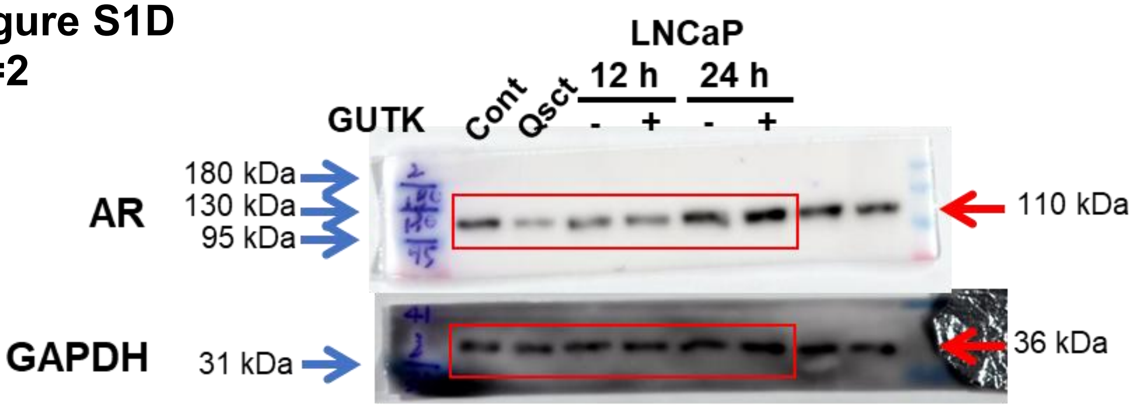

Data S1. Original Western Blot. Full uncropped western blot membranes for all figures in the main text and supplementary information.
